# Current and Future Disease Burden From Ambient Ozone Exposure in India

**DOI:** 10.1029/2018GH000168

**Published:** 2018-11-13

**Authors:** Luke Conibear, Edward W. Butt, Christoph Knote, Dominick V. Spracklen, Stephen R. Arnold

**Affiliations:** ^1^ Engineering and Physical Sciences Research Council (EPSRC) Centre for Doctoral Training (CDT) in Bioenergy University of Leeds Leeds UK; ^2^ Institute for Climate and Atmospheric Science, School of Earth and Environment University of Leeds Leeds UK; ^3^ Meteorological Institute LMU Munich Germany

**Keywords:** ambient air quality, ozone, India, health impact, sources, scenarios, WRF‐Chem

## Abstract

Long‐term ambient ozone (O_3_) exposure is a risk factor for human health. We estimate the source‐specific disease burden associated with long‐term O_3_ exposure in India at high spatial resolution using updated risk functions from the American Cancer Society Cancer Prevention Study II. We estimate 374,000 (95UI: 140,000–554,000) annual premature mortalities using the updated risk function in India in 2015, 200% larger than estimates using the earlier American Cancer Society Cancer Prevention Study II risk function. We find that land transport emissions dominate the source contribution to this disease burden (35%), followed by emissions from power generation (23%). With no change in emissions by 2050, we estimate 1,126,000 (95UI: 421,000–1,667,000) annual premature mortalities, an increase of 200% relative to 2015 due to population aging and growth increasing the number of people susceptible to air pollution. We find that the International Energy Agency New Policy Scenario provides small changes (+1%) to this increasing disease burden from the demographic transition. Under the International Energy Agency Clean Air Scenario we estimate 791,000 (95UI: 202,000–1,336,000) annual premature mortalities in 2050, avoiding 335,000 annual premature mortalities (45% of the increase) compared to the scenario of no emission change. Our study highlights that critical public health benefits are possible with stringent emission reductions, despite population growth and aging increasing the attributable disease burden from O_3_ exposure even under such strong emission reductions. The disease burden attributable to ambient fine particulate matter exposure dominates that from ambient O_3_ exposure in the present day, while in the future, they may be similar in magnitude.

## Key Points

- The disease burden associated with ozone exposure in India is larger using updated risk functions relative to earlier risk functions
- Stringent emission controls offset part of the increasing future disease burden from ozone exposure caused by population aging and growth
- The present‐day disease burden from ambient PM_2.5_ exposure dominates ambient O_3_, while in the future, they may be similar in magnitude

## 1 Introduction

Tropospheric ozone (O_3_) is a secondary gaseous pollutant produced by photochemical oxidation of volatile organic compounds (VOCs) and carbon monoxide (CO) in the presence of nitrogen oxides (NO_*x*_). Increased anthropogenic emissions of O_3_ precursors since the preindustrial have led to large‐scale enhancements in O_3_ throughout the troposphere (Young et al., 2013). Long‐term exposure to ambient O_3_ contributes to the risk of premature mortality (Atkinson et al., 2016; Jerrett et al., 2009; Turner et al., 2016; U.S. Environmental Protection Agency, 2013; World Health Organization, 2013). The Global Burden of Diseases, Injuries, and Risk Factors Study (GBD) in 2016 attributed 233,638 (95% uncertainty interval (95UI): 90,109–385,303) annual premature mortalities to ambient O_3_ exposure globally, with 39% of the disease burden in India (Cohen et al., 2017; GBD 2016 Risk Factors Collaborators, 2017). The GBD2016 used the earlier American Cancer Society Cancer Prevention Study II (CPS‐II) risk estimates (Jerrett et al., 2009) for the cause of chronic obstructive pulmonary disease (COPD) only. These risk estimates have been updated for the same CPS‐II cohort through an extended follow‐up with an expanded study population (Turner et al., 2016). Hazard ratios (HRs) were found to increase (Turner et al., 2016). The updated risks have been used to estimate global premature mortality from long‐term ambient O_3_ exposure, resulting in substantially increased estimates, including in India (Malley et al., 2017). This increased public health burden due to O_3_ exposure has implications for air quality management strategies.

India is rapidly developing where changing energy demand, urbanization levels, socioeconomics, land use, technological choices, and air quality policies are leading to rapid changes in air pollution (Gordon et al., 2014; Health Effects Institute International Scientific Oversight Committee, 2010; International Energy Agency, 2016; King et al., 2013). India has experienced considerable growth in recent years in industrial, power generation, transport, and residential sectors (Sahu et al., 2017). Population‐weighted seasonal ambient O_3_ concentrations in India have increased by 27% from 62 parts per billion (ppb) in 1990 to 77 ppb in 2016 (Health Effects Institute, 2018). O_3_ concentrations have been increasing in India due to increasing emissions of O_3_ precursors (Ghude et al., 2013; Roy et al., 2017).

A business‐as‐usual scenario in India is projected to increase O_3_ precursor emissions and future O_3_ concentrations (Chatani et al., 2014; Fiore et al., 2012; Pommier et al., 2018; Pozzer et al., 2012; Wild et al., 2012), with associated projections increasing the disease burden from O_3_ exposure in India (Lelieveld et al., 2015). Previous studies have predicted an additional smaller increase due to climate change (Kumar et al., 2018; Pommier et al., 2018; Silva et al., 2017). Pommier et al. (2018) analyzed the combined impacts of climate change and emission scenarios on future air quality in South Asia by 2050 and found the substantial increase in anthropogenic emissions in India to have at least a factor of three larger impacts on O_3_ concentrations than the impacts of climate change. Kumar et al. (2018) studied Representative Concentration Pathways (RCP) in South Asia to 2050 and found South Asian daily average 8‐hr O_3_ concentrations changed by +11 ppbv and +2 ppbv by 2050 relative to 2015 under RCP8.5 and RCP6.0, respectively, due to the combined effects of a changing climate and air pollutant emissions. West et al. (2007) found South Asian O_3_ concentrations to increase by 15 ppbv under the Intergovernmental Panel on Climate Change Fourth Assessment Report A2 emission scenario by 2030, where 10% and 35% reductions were obtainable from the current legislation and maximum feasible reduction scenarios, respectively. Silva et al. (2017) estimated the health impacts from climate changes on O_3_ concentrations in India to be 16,000 premature mortalities per year by 2100. This estimate is 18% of the current disease burden in India attributable to ambient O_3_ exposure estimated by the GBD2016 (Cohen et al., 2017; GBD 2016 Risk Factors Collaborators, 2017).

Regional reductions in methane (CH_4_), an important O_3_ precursor, have been found to lower future O_3_ concentrations in India, reducing the exposure associated disease burden (Anenberg et al., 2012). Reductions in non‐methane VOCs (NMVOC) and NO_*x*_ may have immediate air quality and health benefits near the emission reductions (Anenberg et al., 2012; West et al., 2006, 2007). NO_*x*_ reductions in Africa and North America impact Indian O_3_ concentrations in spring, particularly in Delhi, while NO_*x*_ reductions in Southeast Asia and East Asia influence Indian O_3_ concentrations in winter, specifically over Southern India (West et al., 2009a, 2009b). Indian O_3_ concentrations are mostly NO_*x*_‐sensitive, where NO_*x*_ emission reductions reduce the disease burden associated with O_3_ exposure (Anenberg et al., 2009; West et al., 2009a). Regional transport contributes 10% to this reduction in the disease burden (Anenberg et al., 2009).

The contribution of sources to the disease burden from ambient O_3_ exposure in India were estimated in previous global studies (Lelieveld et al., 2015; Silva, Adelman, et al., 2016) using the earlier CPS‐II risk estimates, which found substantial contributions from precursor emissions from energy, land transport, and residential sources. Previous studies of the total or source‐specific disease burden associated with O_3_ exposure have used global, offline chemical transport models at relatively coarse spatial resolution (between 0.5° × 0.67° and 2.0° × 2.5°; Lelieveld et al., 2015; Malley et al., 2017; Silva, Adelman, et al., 2016; Silva, West, et al., 2016). Tropospheric O_3_ has a nonlinear dependence on precursor concentrations, with production on short time scales (Carey Jang et al., 1995; Liang & Jacobson, 2000; Sharma & Khare, 2017; Wild & Prather, 2006). Coarse spatial resolution models dilute O_3_ precursors, causing simulated concentrations to diverge from observations (Carey Jang et al., 1995; Liang & Jacobson, 2000; Sharma & Khare, 2017; Wild & Prather, 2006). Model resolution also affects estimates of the O_3_ exposure‐related disease burden (Punger & West, 2013; Thompson & Selin, 2012; Thompson et al., 2014). Online‐coupled modeling explicitly accounts for feedbacks between chemistry and meteorology (Baklanov et al., 2014; Grell et al., 2004), which can be important to consider when emissions are changing.

Here we make the first estimate of the source‐specific disease burden due to O_3_ exposure at high spatial resolution, using the updated CPS‐II risk functions from Turner et al. (2016), in addition to analyzing the impacts from future air pollution control pathways. We use a high‐resolution (30 km, 0.3° spatial resolution) numerical weather prediction model, online‐coupled with chemistry, to estimate the present‐day reductions in surface O_3_ concentrations in India resulting from the removal of different O_3_ precursor source sectors. An annual control simulation is performed for the year 2014 and evaluated against surface observations, then annual sensitivity simulations are performed individually removing emissions from biomass burning (BBU), power generation (ENE), industrial nonpower (IND), residential energy use (RES), and land transport (TRA). We explore the impact of scenarios conducted in line with the International Energy Agency (IEA) New Policy Scenario (NPS) and Clean Air Scenario (CAS) (International Energy Agency, 2016). To help interpret the impacts of these emission scenarios, we conduct idealized simulations where we individually change emissions (−10% and +10%) for the four anthropogenic emission sectors (ENE, IND, RES, and TRA). We calculate the associated disease burden per risk estimate, per simulation. We then perform further sensitivity studies to explore the impacts of the Indian demographic and epidemiologic transition through to 2050 on the public health burden associated with O_3_ exposure. We assume that both climate and emissions from countries outside India remain unchanged, allowing us to isolate the impacts of changing Indian emissions. We aim to produce a valuable resource to help inform environmental policy decisions at the state and national levels regarding O_3_ precursor emission controls.

## 2 Methods

### 2.1 Model Description

**Figure 1:**
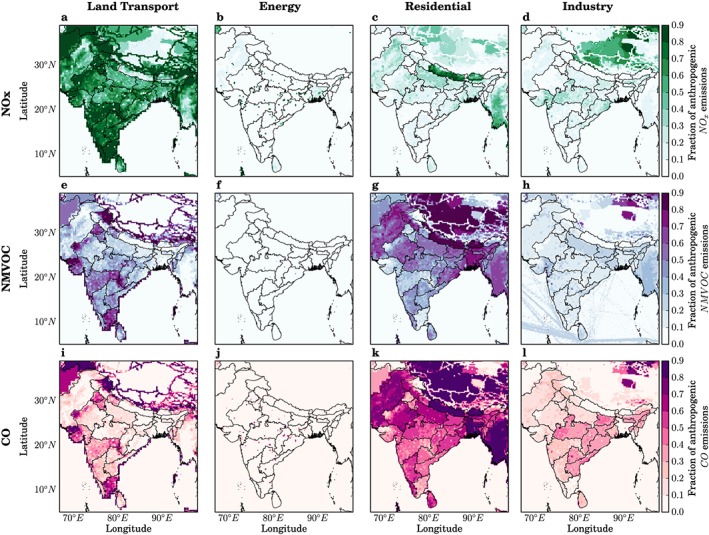
Fractional contribution per sector to total annual anthropogenic emissions. Fractional contribution of land transport (TRA), power generation (ENE), residential energy use (RES), and industry (IND) to anthropogenic emissions of (a–d) nitrogen oxides (NO_*x*_), (e–h) nonmethane volatile organic compounds (NMVOC), and (i–l) carbon monoxide (CO).

We use the online‐coupled Weather Research and Forecasting model coupled with Chemistry (Grell et al., 2005) version 3.7.1 (National Center for Atmospheric Research et al., 2015). The model setup, emission inventories, and model evaluation are described in detail by Conibear et al. (2018a) and are summarized in Table S1 in the supporting information. The model domain covers South Asia at 30 km (0.3°) horizontal resolution. Anthropogenic emissions are from the Emission Database for Global Atmospheric Research with Task Force on Hemispheric Transport of Air Pollution (EDGAR‐HTAP) version 2.2 (Janssens‐Maenhout et al., 2015) for 2010 at 0.1° × 0.1° horizontal resolution. Power generation emissions for the energy sector are from electricity and heat production. Industrial nonpower emissions include large‐scale combustion and industrial processes. Residential energy use is defined by small‐scale combustion including heating, cooking, lighting, cooling, and auxiliary engines. Figure 1 shows the fractional contribution of land transport, power generation, residential energy use, and industry to total annual anthropogenic emissions of NO_*x*_, NMVOC, and CO. Land transport dominates anthropogenic emissions of NO_*x*_, while residential energy use is the leading contributor to anthropogenic emissions of CO and NMVOC. Anthropogenic emissions of NO_*x*_, NMVOC, and CO are all highest during the winter (DJF) and lowest in the summer (JJA) (Figure S1 in the supporting information).

Biomass burning emissions are from the Fire Inventory from NCAR (FINN) version 1.5 (Wiedinmyer et al., 2011). Biomass burning emissions include the open burning of biomass including wildfires, agricultural fires, and prescribed fires, and not biofuel use and trash burning (Wiedinmyer et al., 2011). Small agricultural fires are challenging to detect by satellite (Randerson et al., 2012). FINN better resolves emissions from these small fires compared to some other fire emission data sets (Reddington et al., 2016). However, it is likely that emissions from agricultural fires are still underestimated in our study (Cusworth et al., 2018). Biomass burning emissions are largest in spring (MAM) and late autumn (ON), due to open crop residue burning postharvesting season, and smallest in the summer (JJA) (Venkataraman et al., 2006). The Model of Emissions of Gases and Aerosol from Nature (Guenther et al., 2006) calculates biogenic emissions online. The Global Ozone Chemistry Aerosol Radiation and Transport with Air Force Weather Agency modifications (Chin et al., 2000) calculates dust emissions online.

Gas phase chemistry is simulated using the Model for Ozone and Related Chemical Tracers, version 4 (MOZART‐4; Emmons et al., 2010) with several updates to aromatic photochemistry, biogenic hydrocarbons, and other species relevant to regional air quality (Hodzic & Jimenez, 2011; Knote et al., 2014). The Model for Simulating Aerosol Interactions and Chemistry scheme (MOSAIC, Zaveri et al., 2008) with the simplified description of organic aerosols from Hodzic and Jimenez (2011) calculates aerosol physics and chemistry with four sectional discrete size bins: 0.039–0.156 μm, 0.156–0.625 μm, 0.625–2.5 μm, and 2.5–10 μm (Hodzic & Knote, 2014). Photolysis rates are calculated with the Fast Tropospheric Ultraviolet‐Visible module (Tie et al., 2003), and radiation is calculated with the Rapid Radiative Transfer Model (Iacono et al., 2008).

### 2.2 Model Evaluation

We evaluate model simulated O_3_ using surface measurements from previous measurement studies over India representative of different chemical environments. Details of the monitoring sites are in Table S2. Surface O_3_ observations used ultraviolet photometry online analyzers with a 5% accuracy (Kleinman et al., 1994). Similar to previous studies, the model evaluation uses observational data of a different year to the simulation due to the paucity of O_3_ observations over India (Kumar, Naja, Pfister, Barth, & Brasseur, 2012; Kumar, Naja, Pfister, Barth, Wiedinmyer, et al., 2012; Kumar et al., 2015, 2018; Ojha et al., 2016; A. Sharma, Ojha, et al., 2017).

**Figure 2:**
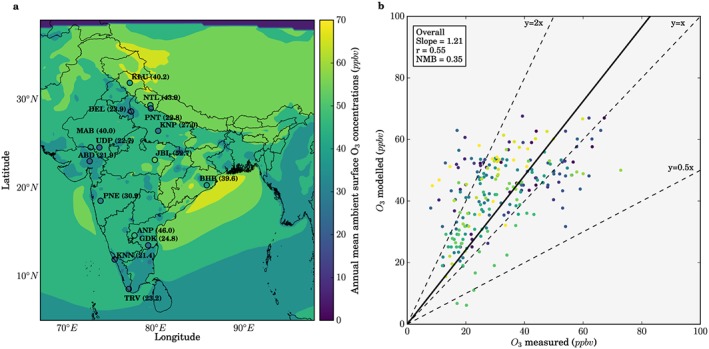
Comparison of observed and simulated O_3_ concentrations. (a) Annual mean surface O_3_ concentrations from the model for 2014 (background) compared with observations (filled circles, with text showing site abbreviation and measured annual mean). (b) Comparison of annual and monthly mean surface O_3_ concentrations (the colors of filled circles are grouped per site for annual and monthly values). We show the overall best fit line as solid, and the 1:1, 2:1, and 1:2 lines as dashed lines. Comparison of annual mean observed and simulated values: normalized mean bias (NMB) = 0.35, the best fit line has slope = 1.21, and Pearson's correlation coefficient (*r*) = 0.55. Separate comparison against rural sites: NMB = 0.28, slope = 1.18, and *r* = 0.67. Urban sites: NMB = 0.41, slope = 1.24, and *r* = 0.47.

Figure 2 shows the comparison of observed and simulated O_3_ concentrations. Simulated O_3_ concentrations are highest in northern and eastern India. Simulated O_3_ concentrations are lowest in summer (JJA), as seen in previous studies (Chatani et al., 2014; Lu et al., 2018; Ojha et al., 2016; S. Roy et al., 2008). The low O_3_ concentrations in summer are likely due to the monsoon cloud cover reducing solar radiation and wet scavenging of O_3_ precursors suppressing O_3_ production (Lu et al., 2018). Our simulations overestimate surface O_3_ concentrations relative to observations with an overall normalized mean bias (NMB) of 0.35. Previous modeling studies over India also mostly overestimate O_3_ relative to observations (Chatani et al., 2014; Engardt, 2008; Gupta & Mohan, 2015; Karambelas et al., 2018; Kota et al., 2018; Kumar et al., 2010; Kumar, Naja, Pfister, Barth, & Brasseur, 2012; Kumar, Naja, Pfister, Barth, Wiedinmyer, et al., 2012; Lu et al., 2018; Marrapu et al., 2014; Ojha et al., 2012, 2016; Pommier et al., 2018; S. Roy et al., 2008; A. Sharma, Ojha, et al., 2017; S. Sharma et al., 2016; Surendran et al., 2015).

Models have been found to have limited success in simulating local scale titration chemistry of O_3_ with nitric oxide (NO), causing O_3_ concentrations to be overestimated (Chatani et al., 2014; Engardt, 2008; Pommier et al., 2018; S. Sharma et al., 2016; Sharma, Sharma, et al., 2017). Urban observations close to large NO_*x*_ emission sources tend to have low O_3_ concentrations due to this titration effect (Clapp & Jenkin, 2001). To examine this effect, we separately evaluate the model against rural (including semirural, coastal, and high altitude) and urban (including semiurban) observations (Figure S2). The model simulates O_3_ concentrations closer to observations for rural sites (NMB = 0.28) than for urban sites (NMB = 0.41). This suggests that the titration of O_3_ with large NO concentrations may be causing the model to overestimate O_3_ where high NO concentrations are likely not captured by the model.

Previous studies have found Indian O_3_ concentrations to be larger downwind of heavily populated regions relative to urban areas (Lal et al., 2008; Lawrence & Lelieveld, 2010). The combination of EDGAR‐HTAP with MOZART has also been found to enhance surface O_3_ mixing ratios due to vertical mixing of enhanced O_3_ that has been produced aloft (S. Sharma et al., 2016). The overestimation of O_3_ could also be due to our underestimation of dust aerosols (Conibear et al., 2018a). Heterogeneous reactions on dust surfaces have been found to reduce O_3_ concentrations (Kumar et al., 2014; Li et al., 2017). Accurate simulation of dust emissions and including these heterogeneous reactions reduced the difference between observed and simulated O_3_ concentrations in India from 16 to 2 ppbv (Kumar et al., 2014). Model overestimation of O_3_ could also be due to uncertainties in emission inventories over India (Janssens‐Maenhout et al., 2015; Jena et al., 2015; Monks et al., 2015; Saikawa et al., 2017).

Overall, our simulation of O_3_ concentrations is similar to previous studies (Chatani et al., 2014; Gupta & Mohan, 2015; Karambelas et al., 2018; Kota et al., 2018; Kumar et al., 2018; Kumar, Naja, Pfister, Barth, & Brasseur, 2012; Kumar, Naja, Pfister, Barth, Wiedinmyer, et al., 2012; Pommier et al., 2018; A Sharma, Sharma, et al., 2017; S Sharma et al., 2016), and we compare concentrations in this control (CTL) simulation to that simulated under a number of different air pollution control pathways (section 2.3).

### 2.3 Air Pollution Control Pathways

We explore the sensitivity of surface O_3_ concentrations and the resultant disease burden to different air pollution control pathways (scenarios) by conducting annual simulations using meteorology and boundary conditions for the year 2014. These scenarios are discussed in detail in Conibear et al. (2018b). Briefly, we scale the anthropogenic emissions from the control scenario by factors from the NPS and the CAS from the IEA (International Energy Agency, 2016). The NPS considers all relevant existing and planned policies as of 2016, while the CAS represents stringent and proven energy policies and technologies tailored to national circumstances. By 2040 in India, the NPS reduces the growth of sulfur dioxide (SO_2_), NO_*x*_, and fine particulate matter (PM_2.5_) to +9% on average relative to 2015 emissions, while the CAS brings emissions of SO_2_, NO_*x*_, and PM_2.5_ below 2015 levels by an average of 65%. To help interpret the results for these scenarios, we conduct idealized simulations where each of the anthropogenic sectors (ENE, IND, RES, and TRA) has emissions increased or decreased by 10%. These anthropogenic sectors have been previously found to be key sources of O_3_ precursors (S. Sharma et al., 2016; Silva, Adelman, et al., 2016; Silva, West, et al., 2016). These are in addition to the simulations where we individually remove the emissions from BBU, ENE, IND, RES, and TRA sectors.

### 2.4 Health Impact Estimates

Long‐term exposure to O_3_ has been found to be a likely cause of detrimental respiratory effects (U.S. EPA, 2013). We do not estimate the impact of long‐term O_3_ exposure on cardiovascular mortality due to limited causal evidence (U.S. EPA, 2013). We calculate the disease burden associated with COPD from ambient O_3_ exposure using relative risk (RR) estimates from the earlier CPS‐II study (Jerrett et al., 2009), in addition to the updated CPS‐II study (Turner et al., 2016). The updated CPS‐II study derived RR estimates from a larger study population (+49%), studying twice as many deaths during a longer follow‐up period (+22%). The updated CPS‐II study used improved exposure estimates and found the HRs for respiratory mortality increased. The earlier CPS‐II study found HR per 10 ppb for respiratory mortality after adjusting for PM_2.5_ confounding of 1.04 (95UI: 1.01–1.07), while the updated CPS‐II study found HR for COPD mortality after adjusting for PM_2.5_ and nitrogen dioxide (NO_2_) confounding of 1.14 (95UI: 1.08–1.21). The updated CPS‐II study found through sensitivity analyses that the long‐term O_3_ health impacts are not confounded by socioeconomic status or modeling approach. To be consistent with the GBD, we estimate premature mortality from the risk of ambient O_3_ exposure from the cause of COPD only. The GBD used the earlier CPS‐II study risks with 3‐month average daily maximum 1‐hr O_3_ concentrations (3mDMA1), while the updated CPS‐II study used annual average daily maximum 8‐hr O_3_ concentrations (ADM8h). Here we use both the earlier (Jerrett et al., 2009) and updated (Turner et al., 2016) RR estimates, with the corresponding O_3_ metric.

**Figure 3:**
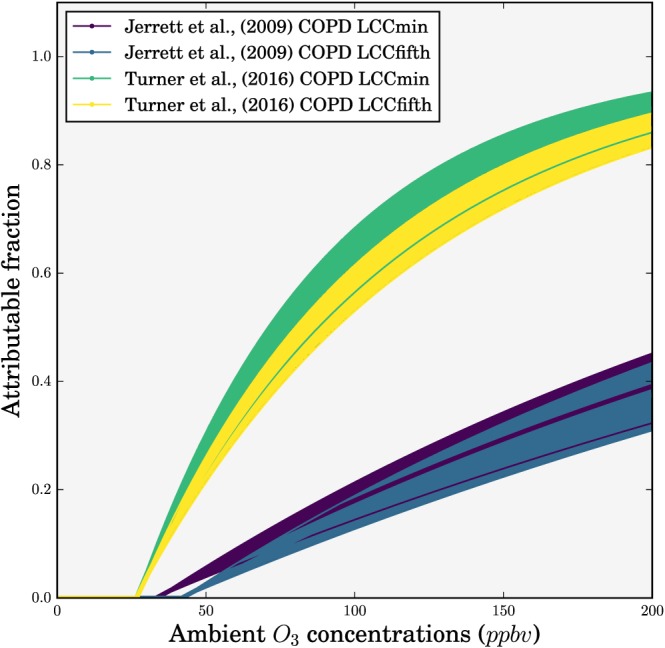
Attributable fractions as a function of ambient O_3_ concentrations for chronic obstructive pulmonary disease from both the earlier American Cancer Society Cancer Prevention Study II (CPS‐II; Jerrett et al., 2009) and the updated CPS‐II study (Turner et al., 2016). Mean (solid line) as well as upper and lower 95% confidence intervals (shading) shown for both low‐concentration cutoffs (LCC_min_ and LCC_fifth_).

We estimate premature mortality associated with O_3_ exposure (M) for COPD for adults over 25 years of age (as per GBD) following equation (1). Mortality is a function of the baseline mortality rate (I), attributable fraction (AF), and the exposed population (P) per age group. AF is a function of the effect estimate (β) and the change in O_3_ concentrations (ΔX) relative to the low‐concentration cutoff (LCC), given in equation (2). Both the earlier and updated CPS‐II study AF functions are given in Figure 3, clearly showing the impact of the increased HR for the updated CPS‐II study. The exposure‐response function for the updated CPS‐II is nonlinear, relative to the earlier CPS‐II function, where there are larger changes in risk for low concentrations compared with higher concentrations (Pope et al., 2015).

Two LCCs represent uncertainty in the HR as either the minimum exposure (LCC_min_) or the fifth percentile (LCC_fifth_), whereby if the O_3_ concentration is below the LCC, there is no effect of O_3_ exposure on mortality and ΔX equals zero. The earlier CPS‐II study (Jerrett et al., 2009) used the minimum and fifth percentile LCCs of 33.3 and 41.9 ppb, respectively, while the updated CPS‐II study (Turner et al., 2016) used the minimum and fifth percentile LCCs of 26.7 and 31.1 ppb, respectively. Epidemiological studies generally find little evidence for low concentration thresholds, and disease burden estimates using thresholds will therefore be conservative (U.S. EPA, 2013). β is the natural log of the HR for a 10‐ppb increase in long‐term O_3_ exposure (equation (3).

(1) M=I×AF×P

(2) $$\mathrm{AF}=1-e^{\mathrm{\beta \Delta X}}$$

(3) $$\beta =\frac{\ln\left(\mathrm{HR}\right)}{10}$$

Years of life lost (YLL) are estimated following equation (4), where the premature mortality (*M*) is multiplied by the age‐specific life expectancy (LE) remaining at the age of death from the standard reference life table from GBD2016 (Global Burden of Disease Study 2016, 2017).

(4) YLL=M×LE

### 2.5 Future Demographics and Baseline Mortality Rates in India

Population density, age groupings, and baseline mortality rates are taken from the International Futures (IFs) integrated modeling system (Hughes et al., 2011) baseline scenario (Hughes et al., 2012) for 2015 and 2050. In Conibear et al. (2018b), we found that the population age distribution in India in 2050 shifts toward older ages relative to 2015 (40 years and older) and there is large population growth, particularly across the Indo‐Gangetic Plain. The baseline mortality rate for COPD reduces slightly in 2050 relative to 2015 for age groupings 60 years and older. The baseline mortality rate for COPD in 2015 from IFs is slightly larger than the corresponding rate from GBD2016 for age groupings 75 years and older, and lower than those from GBD2015 (Institute for Health Metrics and Evaluation, 2018). The Gridded Population of the World, Version 4 national identifier grid (Center for International Earth Science Information Network, & NASA Socioeconomic Data and Applications Center, 2016) allocated data by country.

### 2.6 Uncertainties and Assumptions

To account for uncertainty in the RR estimates, we sample 1,000 estimates of β from normal distributions of β using 95% uncertainty intervals to derive a distribution of the AF. We estimate the uncertainty in O_3_ concentrations for each metric (3mDMA1 and ADM8h) per grid cell as ±2 standard deviations using daily O_3_ concentrations from the metric value. We combine the fractional errors in quadrature (i.e., square root of the sum of squares). Emission inventories of important O_3_ precursors, such as NO_*x*_ emissions, are uncertain over India (Janssens‐Maenhout et al., 2015; Jena et al., 2015; Monks et al., 2015; Saikawa et al., 2017). We only use a single model to estimate O_3_ concentrations, while previous studies have highlighted the importance of using an ensemble of estimates (Post et al., 2012; Silva, Adelman, et al., 2016; Silva, West, et al., 2016). Overestimated O_3_ production in polluted regions may bias the source attribution of O_3_ more toward local sources due to the shorter production time scales (Wild & Prather, 2006). The response of O_3_ concentrations to changes in emissions is nonlinear, meaning an emission removal approach to quantify attributions can differ from a source tracking approach (Clappier et al., 2017; Mertens et al., 2018).

We use the same meteorology for all simulations to focus on the impacts of emission changes and hence do not include the impacts of climate changes on O_3_, although these climate‐driven changes are likely smaller relative to those driven by emission changes (Kumar et al., 2018; Pommier et al., 2018; Silva et al., 2017). Our results are limited to the impacts from these projected emission changes in India on the disease burden associated with O_3_ exposure in the context of changing demographics and background mortality rates. Reductions in O_3_ precursors in India may reduce O_3_ concentrations outside of India, providing public health benefits not accounted for in our study (West et al., 2009a). Reduced O_3_ concentrations will also reduce damage to crops and the economic cost associated with premature mortalities (Ghude et al., 2014, 2016; Sinha et al., 2015), as well as providing substantial climate co‐benefits (Shindell et al., 2012).

We do not consider the short‐term health impacts of ambient O_3_ exposure (WHO, 2013). The risk estimates we use for O_3_ have been adjusted for confounding from PM_2.5_ and NO_2_, implying that double counting of health impacts from PM_2.5_ and O_3_ exposure should be small. However, we avoid summing the disease burden from ambient O_3_ and PM_2.5_ exposure as they are caused by similar diseases (e.g., COPD). We assume that the risk estimates from the CPS‐II study apply to the Indian population, even though the O_3_ concentrations that the Indian population are exposed to are higher than exposures used in the CPS‐II studies (Malley et al., 2017).

## 3 Results

### 3.1 Comparison of Disease Burden Using earlier and Updated Exposure‐Response Functions

**Figure 4:**
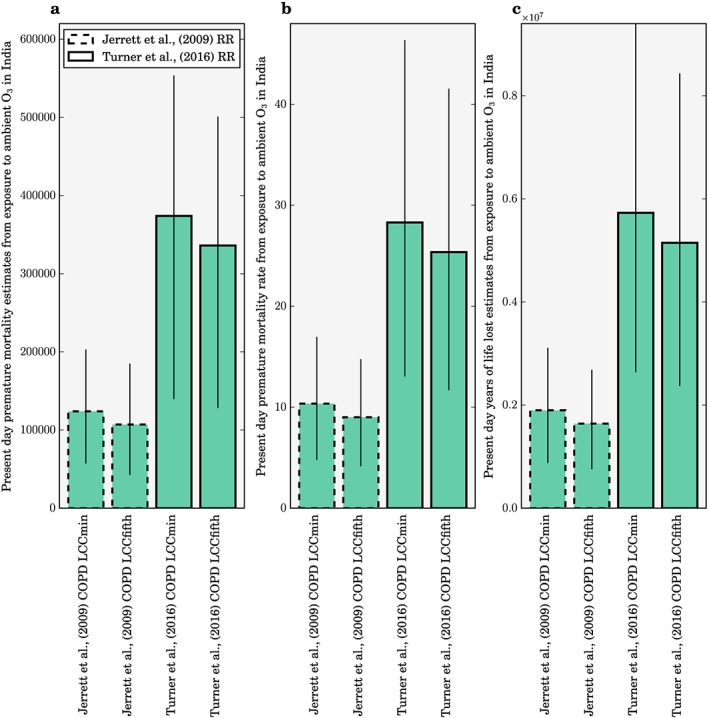
Estimates of (a) premature mortality, (b) mortality rate per 100,000 population, and (c) years of life lost in 2015 from ambient O_3_ exposure in India using the earlier American Cancer Society Cancer Prevention Study II (CPS‐II; Jerrett et al., 2009; dashed bars) and the updated CPS‐II study (Turner et al., 2016; solid bars) risk estimates. The error bars represent 95% uncertainty intervals.

Figure 4 compares the estimates of premature mortality, mortality rate per 100,000 population, and YLL in 2015 from ambient O_3_ exposure in India using our model‐simulated surface O_3_, and risk estimates from both the earlier CPS‐II study (Jerrett et al., 2009) and the updated CPS‐II study (Turner et al., 2016). The disease burden estimates (premature mortality, YLL, and mortality rate) using the updated CPS‐II risks from Turner et al. (2016) are approximately 200% larger than the disease‐burden estimates using the earlier CPS‐II risks from Jerrett et al. (2009). This is primarily due to the larger hazard risk ratios (HR = 1.14 compared to HR = 1.04). Using earlier CPS‐II risks, we estimate 124,000 (95UI: 57,000–203,000) and 107,000 (95UI: 42,000–185,000) annual premature mortalities from COPD due to O_3_ exposure in India for 2015 using LCC_min_ and LCC_fifth_, respectively. Using updated CPS‐II risks, these increase to 374,000 (95UI: 140,000–554,000) and 336,000 (95UI: 128,000–501,000) annual premature mortalities from COPD due to O_3_ exposure in India for 2015 using LCC_min_ and LCC_fifth_, respectively. The annual premature mortality estimates using updated CPS‐II risks are 202% and 214% larger than mortality estimates using earlier CPS‐II risks for LCC_min_ and LCC_fifth_, respectively. We quantify 1,899,000 (95UI: 875,000–3,111,000) and 1,639,000 (95UI: 650,000–2,834,000) annual YLL from COPD due to O_3_ exposure in India for 2015 for earlier CPS‐II risks using LCC_min_ and LCC_fifth_, respectively. These increase to 5,729,000 (95UI: 2,138,000–8,482,000) and 5,148,000 (95UI: 1,963,000–7,679,000) annual YLL from COPD due to O_3_ exposure in India for 2015 for updated CPS‐II risks using LCC_min_ and LCC_fifth_, respectively. The annual YLL estimates using updated CPS‐II risks are 217% and 214% larger than estimates using earlier CPS‐II risks for LCC_min_ and LCC_fifth_, respectively. We estimate that the annual mean mortality rate per 100,000 population for India from O_3_ exposure is 10 (95UI: 4–16) and 8 (95UI: 3–14) for the earlier CPS‐II risks for LCC_min_ and LCC_fifth_, respectively. The mortality rate is 28 (95UI: 11–43) and 25 (95UI: 10–39) per 100,000 population for the updated CPS‐II risks for LCC_min_ and LCC_fifth_, respectively. The annual mortality rates per 100,000 population are 180% and 213% larger for the updated CPS‐II risks relative to the earlier CPS‐II risks for LCC_min_ and LCC_fifth_, respectively.

**Figure 5:**
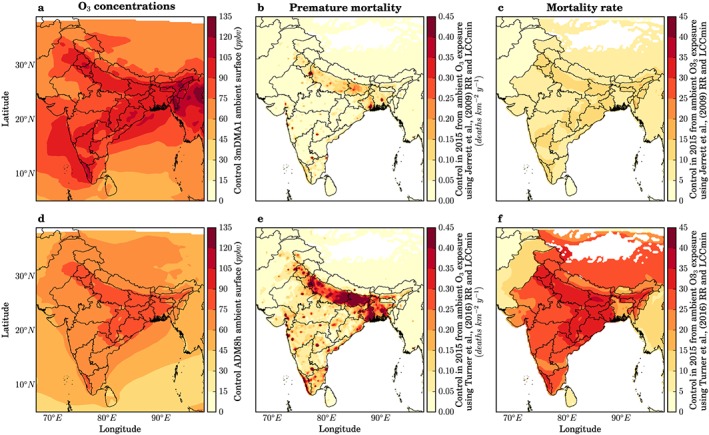
Premature mortality estimates due to O_3_ exposure across India in 2015. All calculations for the control scenario. (a and d) Annual mean surface O_3_ concentrations. (b and e) Annual premature mortality. (c and f) Annual mortality rate per 100,000 population. (a–c) Calculated following Jerrett et al. (2009). (d–f) Calculated following Turner et al. (2016). (a) shows the 3mDMA1 O_3_ metric. (d) shows the ADM8h O_3_ metric. See text for details.

Figure 5 shows the 3mDMA1 and ADM8h O_3_ concentrations across India with the associated respective premature mortality estimates and mortality rate per 100,000 population. The population‐weighted surface O_3_ concentrations for the 3mDMA1 metric is 94.5 ppbv and for the ADM8h metric is 77.2 ppbv. Surface O_3_ concentrations are larger over northern and eastern India. The overall disease burden is highly concentrated in the Indo‐Gangetic Plain, with hot spots across India relating to population density. The mortality rate is less spatially variable, though highest in north eastern India, with values of 13 per 100,000 population for the Jerrett et al. (2009) risks and 34 per 100,000 population for the updated risks from Turner et al. (2016).

### 3.2 Reduction in O_3_ Concentrations and Disease Burden per Source Removal

Table 1 shows the reduction in population‐weighted 3mDMA1 and ADM8h O_3_ concentrations across India associated with the removal of different emission sectors. The spatial changes in annual‐mean surface O_3_ concentrations of these source removals are shown in Figure S3. We find that emissions from land transport dominate (28% of 3mDMA1 and 35% of ADM8h) the reduction in O_3_ concentrations from all individual source removals, with substantial reductions from energy (14% of 3mDMA1 and 23% of ADM8h) and residential energy use emissions (9% of 3mDMA1 and 11% of ADM8h). The summation of these source reductions is 60% from 3mDMA1 and 76% from ADM8h. The summation of source reductions is less than 100% due to sources not investigated in this study (e.g., aircraft NO_*x*_ and biogenic VOCs), sources outside of the domain, natural sources (e.g., stratospheric O_3_ transport, lightning NO_*x*_, and soil NO_*x*_), and due to the nonlinear response of O_3_ to precursor emission changes. The source summation less than 100% has been seen in previous studies over India by Silva, Adelman, et al. (2016), Silva, West, et al. (2016), and S. Sharma et al. (2016), and Fiore et al. (2004) demonstrated the influence of additional sources to O_3_ concentrations in the United States.

**Table 1:** Reduction in Population‐Weighted Surface O_3_ Concentrations in India Associated With Removing Different Sources

Reduction to population‐weighted surface O_3_ concentration		BBU	ENE	IND	RES	TRA	Total
3mDMA1 O_3_	ppbv	4.0	13.5	4.5	8.1	26.4	56.5
	%	4	14	5	9	28	60
ADM8h O_3_	ppbv	1.7	17.4	3.9	8.1	27.0	58.1
	%	2	23	5	11	35	76

*Note*. Two different O_3_ metrics (3mDMA1 and ADM8h) are shown. Sources are biomass burning (BBU), power generation (ENE), industrial nonpower (IND), residential energy use (RES), and land transport (TRA). Absolute (ppbv) and relative (%) reductions are shown.

Table 2 shows the source contributions to the annual premature mortality estimate associated with O_3_ exposure in India in 2015 using Turner et al. (2016) risks and LCC_min_. We calculate the source contributions using two different methods: the attribution and subtraction methods (Conibear et al., 2018a; Kodros et al., 2016). The attribution method estimates the source contribution to the disease burden as the total disease burden estimate multiplied by the fractional source contribution to total O_3_ concentrations from each emission removal simulation. The subtraction (also known as zero‐out) method calculates the difference between the total disease burden estimate and an estimate where a source has been removed. The estimates from the attribution and subtraction method are expected to produce different results due to the shape of the exposure‐response function, despite both methods using the same O_3_ concentrations contributions from each emission removal simulation. We recently showed a similar effect for PM‐related mortality in India (Conibear et al., 2018a).

**Table 2:** Source Contributions to the Annual Premature Mortalities Associated With Ambient O_3_ Exposure in India in 2015 Calculated Using Turner et al. (2016) Risks and LCC_min_, Including Source Contributions to the Annual Premature Mortalities Associated With Ambient PM_2.5_ Exposure From Conibear et al. (2018a)

Source contribution to annual premature mortalities		BBU	ENE	IND	RES	TRA	Total
O_3_ attribution	Number (×10^3^)	7 (3–11)	86 (32–127)	19 (7–28)	41 (15–61)	131 (49–194)	284 (106–421)
	%	2	23	5	11	35	76
O_3_ subtraction	Number (×10^3^)	10 (4–15)	105 (39–156)	22 (8–33)	45 (17–67)	173 (65–256)	355 (133–527)
	%	3	28	6	12	46	95
PM_2.5_ subtraction	Number (×10^3^)	12 (8–16)	90 (60–122)	66 (45–90)	256 (162–340)	43 (29–58)	467 (304–626)
	%	1	9	7	26	4	47

*Note*. The absolute number and percentage of total premature mortalities associated with O_3_ exposure in India are shown for two different methods (attribution and subtraction) and for the subtraction method for PM_2.5_ exposure. Sources are biomass burning (BBU), power generation (ENE), industrial nonpower (IND), residential energy use (RES), and land transport (TRA). The values in parentheses represent the 95% uncertainty intervals (95UI).

At the national level, the contribution from land transport emissions to the disease burden calculated using Turner et al. (2016) risks is 35% for the attribution method and 46% for the subtraction method. For energy emissions, the contribution to the total disease burden is 23% and 28% for the attribution and subtraction methods, respectively. The spatial distributions of the dominant contributing sources (land transport and energy emissions) to the disease burden associated with O_3_ exposure in India are shown in Figure S4. The summation of the source contributions to mortality from the attribution method is 284,000 (95UI: 106,000–421,000) annual premature mortalities (76% of control simulation), while for the subtraction method, it is 355,000 (95UI: 133,000–526,000) annual premature mortalities (95% of control simulation). The source contributions to the O_3_ burden from the attribution method are smaller than source contributions from the subtraction method, due to the present‐day O_3_ concentrations experienced in India being in the linear section of the exposure‐response function, and to the high minimum pollutant threshold (26.7 ppb for Turner et al., 2016 risks and LCC_min_).

### 3.3 Impact of Emission Mitigation Scenarios on O_3_ and Associated Disease Burden

**Figure 6:**
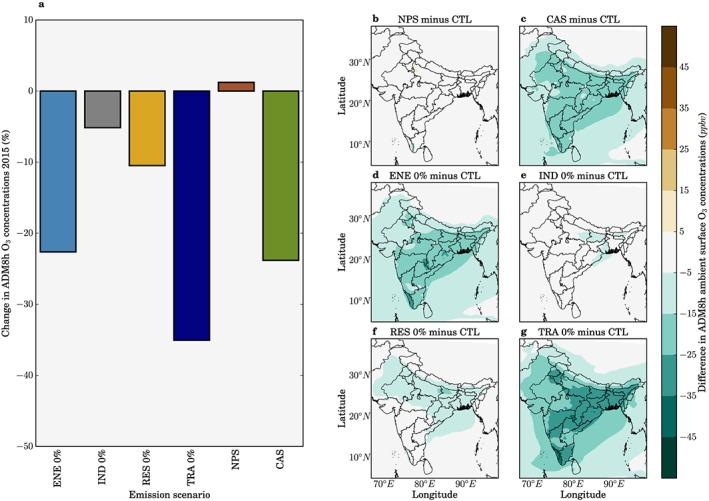
Impacts from air pollution control pathways on surface O_3_ concentrations in India. (a) National‐mean relative changes in population‐weighted ADM8h surface O_3_ concentrations for different emission scenarios relative to the control scenario in 2015. (b–g) Absolute change in ADM8h O_3_ concentrations for different emission scenarios relative to the control (CTL) scenario. Emission scenarios are New Policy Scenario (NPS), Clean Air Scenario (CAS), and when individual emission sectors are switched off: power generation (ENE 0%), industrial nonpower (IND 0%), residential energy use (RES 0%), and land transport (TRA 0%).

Figure 6 shows the impact of different emission scenarios on surface O_3_ concentrations. The NPS increased the population‐weighted ADM8h O_3_ concentrations by 1%, while the CAS reduced them by 24%. The removal of land transport emissions reduces population‐weighted ADM8h O_3_ concentrations by 35%, and the removal of energy emissions reduces them by 23%. These results show that the removal of land transport emissions produces greater reductions in O_3_ concentrations than the CAS. The larger reduction of O_3_ via removing land transport emissions may be due to land transport emissions heavily dominating contributions to anthropogenic NO_*x*_ emissions, coupled to many population regions of India being NO_*x*_ limited. O_3_ production in India has been found to be mostly NO_*x*_‐limited where O_3_ reductions are more sensitive to the emission control of NO_*x*_ than VOC, while some studies find that urban areas are VOC‐limited (Kumar, Naja, Pfister, Barth, & Brasseur, 2012; Kumar, Naja, Pfister, Barth, Wiedinmyer, et al., 2012; Lu et al., 2018; Mahajan et al., 2015; Ojha et al., 2012; Pommier et al., 2018; Saikawa et al., 2017; A. Sharma, Ojha, et al., 2017; S. Sharma et al., 2016). The complex emission changes within the CAS combine a 24% increase in industrial NO_*x*_ emissions and a 78% reduction in land transport NO_*x*_ emissions, with substantial reductions in residential VOC emissions, which can potentially alter the O_3_ sensitivity to NO_*x*_ changes. In the NPS where land transport NO_*x*_ emissions decrease by 45%, O_3_ concentrations increase over Delhi. This increase is possibly due to the urban VOC limited regime where O_3_ production is inversely proportional to NO_*x*_. When designing air pollution control pathways to reduce the health impacts from O_3_ exposure, care is required to consider how changing anthropogenic emissions will affect O_3_ production, and specifically sensitivities to NO_*x*_ and VOC emissions under different regimes.

We estimate the percentage of the population exposed to different concentration levels according to the World Health Organization (WHO) 8‐hr daily maximum O_3_ concentration of 50 ppb, which is the same as the Indian National Ambient Air Quality Standards (NAAQS) released by the Ministry of Environment and Forests, Government of India (Ministry of Environment and Forests, 2009; World Health Organization, 2006). For all scenarios except ENE 0%, CAS, and TRA 0%, all of the Indian populations in both 2015 and 2050 remain exposed to O_3_ concentrations above WHO and NAAQS O_3_ metrics. For the ENE 0%, CAS, and TRA 0% scenarios, 13%, 14%, and 52% of the populations, respectively, had their exposures brought into line with the WHO and NAAQS O_3_ metrics (Figure S5).

**Figure 7:**
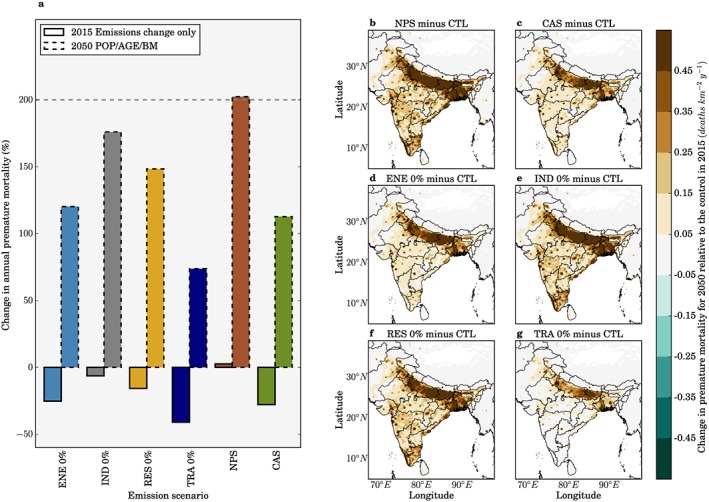
Impacts from air pollution control pathways on annual premature mortality from ambient O_3_ exposure in India. (a) National mean changes in annual premature mortality estimates from ambient O_3_ exposure per scenario, for both emissions only changes between 2015 and 2050 (solid bars) and overall changes in 2050 including 2015 to 2050 changes in emissions as well as population growth, population aging, and baseline mortality rates (POP/AGE/BM, dashed bars). The dashed horizontal line represents the change in annual premature mortality in 2050 if emissions remain at 2015 levels. (b–g) Change in annual premature mortality from ambient O_3_ exposure in 2050 from different emission scenarios (see Figure 6) relative to the control scenario in 2015 accounting for emission changes and POP/AGE/BM changes. All health impacts are calculated using Turner et al. (2016) RR and LCC_min_.

**Figure 8:**
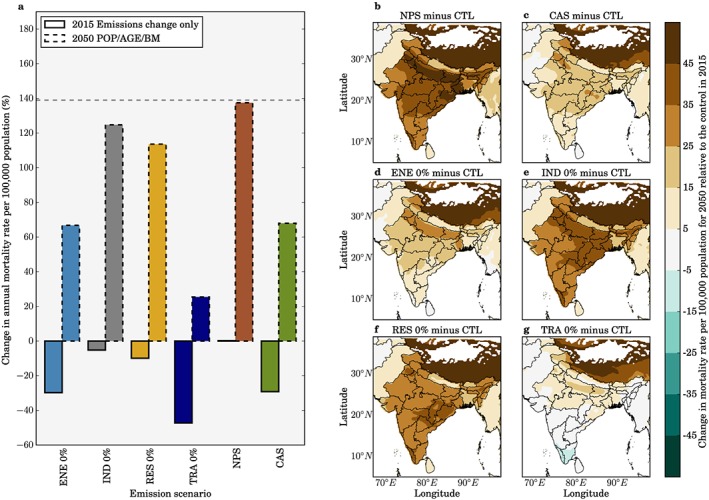
Impacts of air pollution control pathways on annual mortality rate per 100,000 population from ambient O_3_ exposure in India. (a) National mean changes in annual mortality rate per 100,000 population estimates from ambient O_3_ exposure per scenario, for both emissions only changes between 2015 and 2050 (solid bars) and overall changes in 2050 including 2015 to 2050 changes in emissions as well as population aging and baseline mortality rates (POP/AGE/BM, dashed bars). The dashed horizontal line represents the change in annual mortality rate per 100,000 population in 2050 if emissions remain at 2015 levels. (b–g) Annual change in mortality rate per 100,000 population from ambient O_3_ exposure in 2050 from different emission scenarios (see Figure 6) relative to the control scenario in 2015 accounting for emission changes and POP/AGE/BM changes. All health impacts are calculated using Turner et al. (2016) RR and LCC_min_.

Figure 7 shows the impacts of different emission scenarios on total premature mortality associated with O_3_ exposure. Figure 8 shows the corresponding results for the mortality rate per 100,000 population. For emission change only in 2015 (i.e., population, age structure, and background mortality are unchanged), the NPS has small impacts (+1%) on annual premature mortality relative to the control in 2015 due to small change (+1%) in O_3_ in this scenario. In contrast, the CAS reduces annual premature mortality by 30% relative to the control due to the 24% reduction in O_3_ under this scenario. The greater relative reduction in premature mortality compared to O_3_ is due to the strong sensitivity of risk to O_3_ (Figure 3) at the concentrations currently experienced across India (population‐weighted ADM8h estimated to be 77.2 ppbv). The TRA 0% scenario results in a 46% reduction in premature mortality (due to a 35% reduction in O_3_), and the ENE 0% results in a 28% reduction in premature mortality (due to a 23% reduction in O_3_).

The impact of the demographic transition through to 2050 on the premature mortality estimates heavily outweighs the impacts from emission changes. If emissions remain at 2015 levels, we estimate the annual premature mortality will increase by 200% to 1,126,000 (95UI: 421,000–1,667,000) due to population aging and growth increasing the number of people susceptible to air pollution. The impact of the NPS is similar to changes to those from no emission change (+205%). The IND 0% and RES 0% scenarios offset part of the increase in the annual premature mortality estimate due to the demographic transition, avoiding 65,000 (9%) and 136,000 (18%) premature mortalities, respectively. The TRA 0%, CAS, and ENE 0% scenarios offset a substantial amount of the increasing disease burden from the demographic transition, avoiding 520,000 (69%), 335,000 (45%), and 316,000 (42%) premature mortalities per year. This means that even under the stringent emission controls implemented in the CAS, annual premature mortality from ambient O_3_ exposure will increase in 2050 by 111% above the 2015 control scenario to 791,000 (95UI: 202,000–1,336,000) premature deaths.

For no change in emissions through to 2050, the mortality rate per 100,000 population will increase by 139% to 67 (95UI: 24–104) due to the demographic transition. The impact of the NPS by 2050 on mortality rate per 100,000 population is similar to changes to those from no emission change (+139%). The IND 0% and RES 0% scenarios offset part of the increase in the annual mortality rate estimate due to the demographic transition, avoiding 3 (4%) and 7 (10%) annual deaths per 100,000 population, respectively. The TRA 0%, CAS, and ENE 0% scenarios offset 32 (48%), 19 (28%), and 21 (31%) annual deaths per 100,000 population, respectively, from the demographic transition in 2050. Under the stringent emission control scenario of CAS, the annual mortality rate from exposure to O_3_ in 2050 will increase by 71% above the 2015 control scenario to 48 (95UI: 13–86) deaths per 100,000 population.

These results highlight the dominant role of the demographic transition by 2050 in controlling the susceptibility of the Indian population to air pollution, leading to a substantial mortality increase. Stringent air pollution control pathways can provide essential public health benefits offsetting part of the disease burden from O_3_ exposure.

### 3.4 Sensitivities to Demography and Baseline Mortality Rates

To explore the sensitivity of calculated disease burden to different parameters in our methodology, we apply population density from 2015 (POP2015), population age groupings from 2015 (AGE2015), or baseline mortality rates from 2015 (BM2015) individually. Each sensitivity study shows the influence of the other parameters in combination in 2050, highlighting the temporal impact in that specific variable (Figure S6). For the control scenario, the 2015 mortality rate changed by +139% in 2050 due to changes in demography and baseline mortality. In comparison, the mortality rate changed by +96% for POP2015, −21% for AGE2015, and +196% for BM2015. For the control scenario, the 2015 annual premature mortality estimate changed by +201% in 2050 due to changes in demography and baseline mortality. In comparison, premature mortality changed by +134% for POP2015, +2% for AGE2015, and +271% for BM2015. These sensitivity studies highlight the strong dependence of the future disease burden from O_3_ exposure in India to an aging population, where there is a large transition to increased susceptibility.

## 4 Discussion

**Figure 9:**
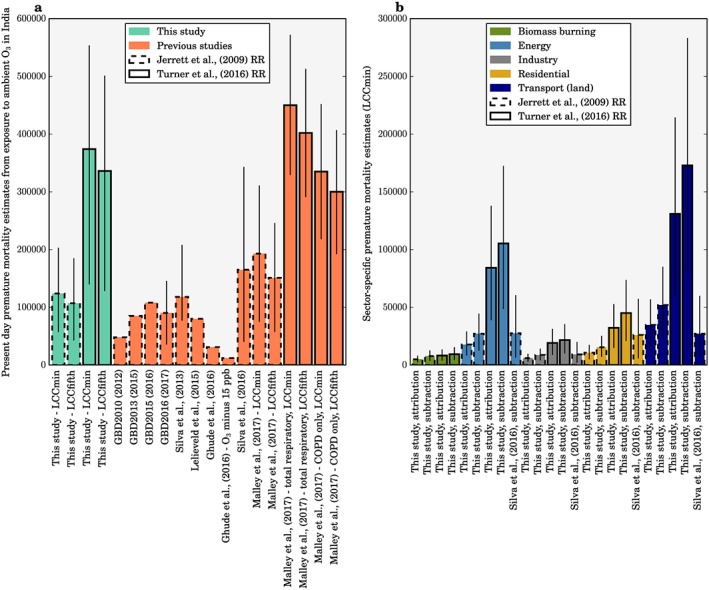
Comparison of premature mortality estimates for India due to ambient O_3_ exposure. Estimates are shown using relative risks from either the earlier American Cancer Society Cancer Prevention Study II (CPS‐II) study from Jerrett et al. (2009; dashed bars) or the updated CPS‐II study from Turner et al. (2016) (solid bars). (a) Total premature mortality from O_3_ exposure from all sources. Estimates shown for both lower concentration cutoffs (LCC_min_ and LCC_fifth_). We compare this study (green) with previous studies (orange). (b) Estimates of premature mortality from different emission sectors (see key) using either the attribution or the subtraction method. Estimates are shown for LCC_min_. The error bars represent 95% uncertainty intervals.

Figure 9 compares our total and source‐specific premature mortality estimates for India from O_3_ exposure with previous studies (Cohen et al., 2017; GBD 2010 Risk Factors Collaborators, 2012; GBD 2013 Risk Factors Collaborators, 2015; GBD 2015 Risk Factors Collaborators, 2016; GBD 2016 Risk Factors Collaborators, 2017; Ghude et al., 2016; Lelieveld et al., 2015; Malley et al., 2017; Silva et al., 2013; Silva, Adelman, et al., 2016; Silva, West, et al., 2016).

For calculations using Jerrett et al. (2009) risks, estimates of the total annual premature mortality from O_3_ exposure in India in the present day vary from 78,000 to 190,000 (GBD 2015 Risk Factors Collaborators, 2016; GBD 2016 Risk Factors Collaborators, 2017; Lelieveld et al., 2015; Malley et al., 2017; Silva et al., 2013; Silva, Adelman, et al., 2016; Silva, West, et al., 2016), between −36% smaller and +55% larger than our estimates. Our estimate of premature mortality is +15% (LCC_min_) and −1% (LCC_fifth_) of the estimate from GBD2015 (GBD 2015 Risk Factors Collaborators, 2016) and +38% (LCC_min_) and +19% (LCC_fifth_) of the estimate from GBD2016 (GBD 2016 Risk Factors Collaborators, 2017). The slightly larger estimates in our study are primarily due to higher estimates of O_3_ concentrations combined with higher (lower) COPD baseline mortality rates from the IFs model relative to GBD2016 (GBD2015). For calculations using Turner et al. (2016) risks, we estimate annual premature mortality to be 12% higher than Malley et al. (2017), the only other study that has applied these risks, due to higher O_3_ concentrations across India in our study. Turner et al. (2016) found significant positive associations between O_3_ and all‐cause, circulatory, and respiratory mortality, suggesting that our estimates from COPD only may be conservative. Overall, our estimates of annual premature mortality in India for the present day from O_3_ exposure are in general agreement with previous key studies.

In terms of source contributions, one previous study calculated the disease burden from O_3_ exposure in India for 2005 using Jerrett et al. (2009) risks and the subtraction method (Silva, Adelman, et al., 2016). For seasonal O_3_ concentrations, they found approximately equal leading contributions from energy (9%), land transport (9%), and residential (9%) emissions. They found that premature mortality fractional contributions were approximately double the fractional contributions to O_3_ concentrations, with 17% from energy, 16% from land transport, and 16% from residential emissions. In contrast, we find land transport to dominate the fractional source contributions to seasonal O_3_ concentrations (28%) over energy (14%) and residential (9%) emissions (Table 1, 3mDMA1 metric). Our larger contribution from land transport may be due to the growth in land transport emissions between 2005 and 2010, where Indian passenger and freight kilometers increased by 6.54% and 3.61% per year, respectively (Venkataraman et al., 2018). In agreement with this previous study, we find the fractional source contributions to premature mortality were approximately double the fractional contributions to O_3_ concentrations due to the low concentration cutoffs, with land transport again dominating (46%) over energy (28%) and residential (12%) emissions (Table 2, subtraction method).

Another study estimated the source contributions to present‐day annual‐mean surface O_3_ concentrations and found land transport emissions to dominate (8%, 3.3 ppb) above those from industry (5%, 2.1 ppb), energy (4%, 1.9 ppb) and residential (3%, 1.4 ppb) sources (S. Sharma et al., 2016). The main difference in source contributions to annual‐mean O_3_ concentrations in our study is that we estimate larger contributions from land transport (30%, 13.6 ppb) and energy (24%, 11.0 ppb) emissions (Figure S3). This is likely due to lower emissions of O_3_ precursors from land transport and energy in the emission inventory used in S. Sharma et al. (2016), relative to that used in our study (EDGAR‐HTAP version 2.2).

Overall, we agree with these previous studies that a number of emission sectors are important, though we find larger contributions from land transport and energy emissions. Future work needs to better constrain the sensitivity of O_3_ concentrations to different emission sectors.

Our results show that decreasing land transport emissions reduces O_3_ concentrations across India. Though in the future with decreasing VOC emissions from the residential sector and increasing the number of vehicles, this trend could change the future sensitivity of O_3_ production to precursor emission changes, which was also found in previous studies (Chatani et al., 2014; Sharma et al., 2016).

Here we have shown that for the disease burden from ambient O_3_ exposure in India, contributions from the subtraction method are up to 43% larger than the attribution method. However, in our previous work estimating the disease burden from PM_2.5_ exposure in India, we showed that the source contributions from the subtraction method are 2–2.5 times smaller than those from the attribution method (Conibear et al., 2018a). In this study for emissions only changes in 2015 for O_3_, the CAS reduces annual premature mortality by 30% relative to the control, due to the 24% reduction in O_3_ under this scenario. In our previous work for emissions only changes in 2015 for PM_2.5_, the CAS reduces annual premature mortality by 39% relative to the control, due to the 67% reduction in PM_2.5_ under this scenario (Conibear et al., 2018b). The relative reduction in premature mortality from O_3_ exposure is larger than the relative reduction in O_3_ concentrations. In contrast, the relative reduction in premature mortality from PM_2.5_ exposure is smaller than the relative reduction in PM_2.5_ concentrations. The exposure‐response functions for both O_3_ and PM_2.5_ are nonlinear. The differences in the relative reductions in concentration and mortality for both PM_2.5_ and O_3_ are due to the position of present‐day pollutant concentrations on the nonlinear health functions, and also due to the different minimum pollutant thresholds assumed, below which there is no health impact.

The O_3_ concentrations currently experienced across India (population‐weighted ADM8h of 77.2 ppbv, Table 1) are in the steeper part of the exposure‐response function, where the health effects are sensitive to changes in pollutant concentrations (Figure 3). In contrast, the PM_2.5_ concentrations currently experienced across India (population‐weighted annual mean of 57.2 μg m^−3^) are in the flatter part of the exposure‐response functions, where the health effects are less sensitive to changing PM_2.5_ concentrations (Conibear et al., 2018a). The O_3_ exposure‐response function assumes that there is no health impact below 26.7 ppbv, the LCC_min_ from Turner et al. (2016). Reducing O_3_ concentrations from the present‐day value (77.2 ppbv) to the LCC_min_, a reduction of 65%, would therefore reduce premature mortality by 100%. The exposure‐response function for PM_2.5_ assumes a theoretical minimum risk exposure level of 2.4 μg m^−3^, meaning that concentrations across India would need to be reduced by 96% (from 57.2 μg m^−3^) to reduce premature mortality from exposure to PM_2.5_ by 100% (Conibear et al., 2018a). This difference results in larger relative reductions in health impacts for the same relative reduction in O_3_, compared to PM_2.5_. If subsequent exposure‐response functions assume different minimum pollutant thresholds (or lower concentration cutoffs, LCC), the calculated sensitivity of health impacts to changes in O_3_ concentrations will also change.

We find that with no emissions change to 2050, the demographic and epidemiological transitions increase the annual premature mortality due to O_3_ exposure by 200% and the mortality rate per 100,000 population by 139%, while the corresponding impacts from PM_2.5_ exposure in India are 75% and 39%, respectively (Conibear et al., 2018b). The larger increase for the O_3_ burden under constant emissions is due to the limited improvement in baseline mortality rate for COPD, while for the PM_2.5_ exposure burden there are substantial reductions in baseline mortality rates for lower respiratory infections, ischemic heart disease, and cerebrovascular disease.

**Figure 10:**
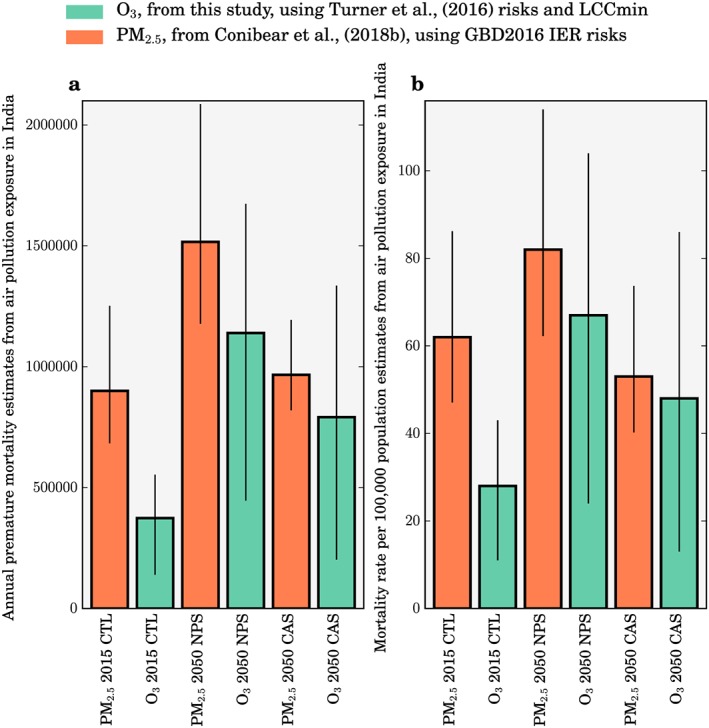
Comparison of (a) premature mortality and (b) mortality rate estimates for India due to PM_2.5_ and O_3_ exposure. Estimates are shown for the 2015 control (CTL) scenario, the New Policy Scenario (NPS) in 2050, and the Clean Air Scenario (CAS) in 2050. Estimates of the disease burden for O_3_ exposure (green) are from this study using relative risks from the updated American Cancer Society Cancer Prevention Study II study from Turner et al. (2016) and LCC_min_. Estimates of the disease burden for PM_2.5_ exposure (orange) are from Conibear et al. (2018b) using the integrated‐exposure response function (Burnett et al., 2014) updated for GBD2016 (GBD 2016 Risk Factors Collaborators, 2017). Estimates for O_3_ exposure are from chronic obstructive pulmonary disease (COPD) only. Estimates for PM_2.5_ exposure are from COPD, lower respiratory infections, cerebrovascular disease, lung cancer, and ischemic heart disease combined. The error bars represent 95% uncertainty intervals.

Figure 10 compares the disease burden from PM_2.5_ and O_3_ exposure in India in 2015 and 2050 due to changes in emissions and changes in demography. In our previous work, we estimated the present‐day disease burden from ambient PM_2.5_ exposure in India to be 900,000 (95UI: 683,000–1,252,000) premature mortalities per year, increasing to 967,000 (95UI: 820,000–1,194,000) under the CAS in 2050, for a 67% reduction in population‐weighted annual mean PM_2.5_ concentrations (Conibear et al., 2018b). Here we estimate the present‐day disease burden from ambient O_3_ exposure in India to be 374,000 (95UI: 140,000–554,000) premature mortalities per year, increasing to 791,000 (95UI: 202,000–1,336,000) under the CAS in 2050, for a 24% reduction in population‐weighted annual mean O_3_ concentrations. This suggests that the future disease burden from PM_2.5_ and O_3_ exposures in India may be similar in magnitude, in contrast to the present day where the disease burden from PM_2.5_ dominates that from O_3_ exposure. This is due to the combination of the smaller reduction in O_3_ (24%) compared to PM_2.5_ (67%) under the CAS and because the COPD baseline mortality rates are predicted to remain high through to 2050, whereas there are substantial reductions in the baseline mortality rates for other diseases that are related to PM_2.5_ exposure (lower respiratory infections, cerebrovascular disease, and ischemic heart disease). The predicted increase in health affects both from PM_2.5_ and O_3_ exposure due to the demographic and epidemiologic changes highlight the challenge facing Indian efforts to reduce the public health risks from exposure to poor air quality in India.

## 5 Conclusion

Long‐term exposure to surface O_3_ is a risk factor for human health in India. Here we are the first to estimate the source‐specific disease burden associated with long‐term O_3_ exposure in India at high spatial resolution, using the updated risk functions from the CPS‐II. We estimate using the updated CPS‐II risk function that in 2015 there were 374,000 (95UI: 140,000–554,000) annual premature mortalities from long‐term O_3_ exposure in India, 200% larger than the disease burden estimates using the older CPS‐II risk function. We find land transport emissions dominate the source contribution to this disease burden (35%), followed by emissions from power generation (23%). The source contributions to the O_3_ disease burden in India from the subtraction method are up to 43% larger than the attribution method due to the position of present‐day O_3_ concentrations on the steeper part of the nonlinear health function and the relatively high minimum pollutant threshold. This is in contrast to the PM_2.5_ disease burden in India where the source contributions from the subtraction method are 2–2.5 times smaller than those from the attribution method due to the position of present‐day PM_2.5_ concentrations on the flatter part of the nonlinear health function and the relatively low minimum pollutant threshold.

With no change in emissions by 2050, we estimate 1,126,000 (95UI: 421,000–1,667,000) annual premature mortalities, an increase of 200% relative to the control in 2015 due to population aging and growth increasing the number of people susceptible to air pollution. We find the IEA NPS provides small changes (+1%) to this increasing disease burden from the demographic transition. Under a stringent air pollution control pathway, the IEA CAS, we estimate 791,000 (95UI: 202,000–1,336,000) annual premature mortalities in 2050. This represents an avoidance of 335,000 premature mortalities a year compared to the scenario of no emission change, offsetting 45% of the increase in premature mortalities due to the demographic transition. However, this shows that even under a scenario of strong emission reductions leading to a 24% reduction in O_3_ concentrations across India, population growth and aging are likely to lead to increasing premature mortality from O_3_ exposure. We do not include impacts of climate change or the impacts of changing emissions from outside India on O_3_ concentrations or the disease burden. We find that the future disease burden from PM_2.5_ and O_3_ exposures in India may be similar in magnitude, in contrast to the present day where the disease burden from PM_2.5_ dominates that from O_3_ exposure. Our study highlights the challenge facing efforts to improve air quality related public health in India, but that critical public health benefits are possible with stringent emission reductions.

## Conflict of Interest

The authors declare no conflicts of interest relevant to this study.

## Supporting information



Supporting Information S1

Data Set S1
