# Supplementary material for: Current and Future Disease Burden From Ambient Ozone Exposure in India
Source: Geohealth. 2018 Nov 13;2(11):334–55. doi: 10.1029/2018GH000168 (PMC7007144; doi:10.1029/2018GH000168)
Supplement: Supplementary file 1 — Supporting Information S1 [file GH2-2-334-s001.pdf]

AGU PUBLICATIONS

*GeoHealth*

Supporting information for

# Current and future disease burden from ambient ozone exposure in India

**Luke Conibear*[1,2], Edward W. Butt[2], Christoph Knote[3], Dominick V. Spracklen[2], and Stephen R. Arnold[2]**

[1] Engineering and Physical Sciences Research Council (EPSRC) Centre for Doctoral Training (CDT) in Bioenergy, University of Leeds, Leeds, UK

[2] Institute for Climate and Atmospheric Science, School of Earth and Environment, University of Leeds, Leeds, UK

[3] Meteorological Institute, LMU Munich, Germany

Corresponding author: Luke Conibear (pmlac@leeds.ac.uk)

## Contents

**Supplementary Table 1:** Model Setup and parameterisation used in the Weather Research and Forecasting model coupled with Chemistry (WRF-Chem) model.

**Supplementary Table 2:** Ambient surface $O_3$ observation site details.

**Supplementary Figure 1:** Fractional contribution per season to total anthropogenic emissions. Fractional contribution to total anthropogenic emissions of NOx from winter (DJF), spring (MAM), summer (JJA), and autumn (SON) to anthropogenic emissions of (a – d) nitrogen oxides (NOx), (e – h) non-methane volatile organic compounds (NMVOC), and (i – l) carbon monoxide (CO).

**Supplementary Figure 2:** Comparison of rural and urban observed and simulated $O_3$ concentrations. (a) Comparison of annual and monthly-mean ambient surface $O_3$ concentrations from rural observation sites. We show the rural site best fit line as solid, and the 1:1, 2:1, and 1:2 lines as dashed. Rural site normalised mean bias (NMB) = 0.28, the rural site best-fit line has slope = 1.18, and rural site Pearson's correlation coefficient (r) = 0.67. (b) Comparison of annual and monthly-mean ambient surface $O_3$ concentrations from urban observation sites. We show the urban site best fit line as solid, and the 1:1, 2:1, and 1:2 lines as dashed. Urban site NMB = 0.41; the urban site best-fit line has slope = 1.24, and urban site r = 0.47.

**Supplementary Figure 3:** Fractional contribution per source to total annual-mean ambient $O_3$ surface concentrations. (a) Total annual-mean ambient $O_3$ surface concentrations. (b – f) Fractional contribution from biomass burning (BBU), power generation (ENE), industrial non-power (IND), residential energy use (RES), and land transport (TRA).

**Supplementary Figure 4:** Dominant source contributions to premature mortality burden due to $O_3$ exposure across India in 2015. (a) Attributable fraction of premature mortalities from land transport emissions (attribution method). (b) Averted fraction of premature mortalities from removing land transport emissions (subtraction method). (c) Attributable fraction of premature mortalities from energy emissions (attribution method). (d) Averted fraction of premature mortalities from removing energy emissions (subtraction method). All health impacts are calculated using Turner et al., (2016) RR and $LCC_{min}$.

**Supplementary Figure 5:** The impact of scenarios on $O_3$ metrics. (a) Percentage of population in 2015 (1st bar) and 2050 (2nd bar) exposed to population-weighted ambient surface $O_3$ concentrations above 50 ppb (WHO AQG, Indian NAAQS) in each scenario. (b) Absolute population in 2015 (1st bar) and

2050 (2nd bar) exposed to population-weighted ambient surface $O_3$ concentrations above 50 ppb (WHO AQG, Indian NAAQS) in each scenario.

**Supplementary Figure 6:** Sensitivities of health impacts due to $O_3$ exposure in India to demography and baseline mortality rates. (a) Mortality rate per 100,000 population. (b) Total annual premature mortality. Impacts are estimated using either Jerrett et al., (2009) (red) and Turner et al., (2016) (purple) relative risks with $LCC_{min}$. For each panel, the control (CTL) scenario is compared against the NPS and CAS scenarios. For each panel, the five bars (left to right) show estimates for 2015 with 2015 population, age, and baseline mortality, 2050 with 2050 population, age, and baseline mortality, and 2050 with population from 2015 (POP2015), population age grouping from 2015 (AGE2015), and baseline mortality rates from 2015 (BM2015).

## Additional Supporting Information (Files uploaded separately)

Supplementary data containing results per Indian state per scenario.

**Supplementary Table 1:** Model Setup and parameterisation used in the Weather Research and Forecasting model coupled with Chemistry (WRF-Chem) model.

| **Model Setup and Parameterisation** | |
|---|---|
| **Process** | **Method** |
| **Domain** | 60° to 100° East, 0° to 40° North |
| **Timestep** | 180 seconds, with Runge-Kutta $2^{nd}$ and $3^{rd}$ order time integration |
| **Horizontal** | Resolution of 30 km along a 140 × 140 grid, with Arakawa C-grid staggering and $2^{nd}$ to $6^{th}$ order advection schemes |
| **Vertical** | 33 vertical levels (top at 10 hPa) with terrain-following hydrostatic pressure coordinates and $2^{nd}$ to $6^{th}$ order advection schemes |
| **Precipitation microphysics** | Thompson scheme (Thompson et al., 2008) |
| **Longwave radiation** | RRTM longwave (Mlawer et al., 1997), called every 30 mins |
| **Shortwave radiation** | RRTM shortwave (Pincus et al., 2003), called every 30 mins |
| **Boundary layer physics** | Mellor-Yamada Nakanishi and Niino 2.5 (Nakanishi et al., 2006), called every timestep |
| **Land surface** | Noah Land Surface Model (Ek et al., 2003) |
| **Convective parameterisation** | Grell 3-D ensemble (Grell et al., 2002), called every 60 seconds |
| **Gas-phase chemistry scheme** | MOZART-4 using KPP (Emmons et al., 2010), chem_opt=201 (Hodzic & Knote, 2014), called every 12 mins |
| **Photolysis scheme** | Madronich fTUV (Tie et al., 2003), called every 30 mins |
| **Aerosol scheme** | MOSAIC 4-bin (Zaveri et al., 2008), called every 12 mins |
| **Dust** | GOCART online with AFWA, dust_opt=3 (Chin et al., 2000, 2002) |
| **Initial & boundary chemistry/aerosol** | MOZART-4 / GEOS5 (The National Center for Atmospheric Research, 2016) |
| **Initial & boundary meteorology** | NCEP GFS and NCEP FNL (NCEP et al., 2000, 2007) |

**Supplementary Table 2:** Ambient surface $O_3$ observation site details.

| Site | Type | Latitude (°N) | Longitude (°E) | Altitude (m) | Data period | Reference |
|---|---|---|---|---|---|---|
| Ahmedabad (ABD) | Semi-arid, urban | 23.00 | 72.60 | 49 | 1993 – 1996 | (Lal et al., 2000) |
| | | | | | 2002 – 2003 | (Sahu & Lal, 2006) |
| | | 23.03 | 72.58 | 53 | 2011 | (Mallik et al., 2015) |
| Anantapur (ANP) | Semi-arid, rural | 14.62 | 77.65 | 331 | 2002 – 2003 | (Reddy et al., 2008) |
| | | | | | 2008 – 2009 | (Reddy et al., 2010) |
| Bhubaneswar (BHB) | Coastal, rural | 20.30 | 85.83 | 45 | 2010 – 2012 | (Mahapatra et al., 2014) |
| Delhi (DEL) | Urban | 28.65 | 77.27 | 220 | 1997 – 2004 | (Jain et al., 2005) |
| Gadanki (GDK) | Rural | 13.50 | 79.20 | 375 | 1993 – 1996 | (Naja et al., 2002) |
| Jabalpur (JBL) | Semi-urban | 23.17 | 79.92 | 411 | 2013 – 2014 | (Sarkar et al., 2015) |
| Kannur (KNN) | Semi-rural | 11.90 | 75.40 | 5 | 2009 – 2010 | (Nishanth et al., 2012) |
| Kanpur (KNP) | Urban | 26.46 | 80.33 | 125 | 2009 – 2013 | (Gaur et al., 2014) |
| Kullu (KLU) | Semi-urban | 31.90 | 77.12 | 1154 | 2010 | (Sharma et al., 2013) |
| Mt. Abu (MAB) | High altitude, rural | 24.60 | 72.70 | 1680 | 1993 – 2000 | (Naja et al., 2003) |
| Nainital (NTL) | High altitude, rural | 29.37 | 79.45 | 1958 | 2006 – 2008 | (Kumar et al., 2010) |
| | | | | | 2009 – 2011 | (Sarangi et al., 2014) |
| Pune (PNE) | Semi-urban | 18.54 | 73.81 | 600 | 2003 – 2004 | (Beig et al., 2007) |
| Pantnagar (PNT) | Semi-urban | 29.00 | 79.50 | 231 | 2009 – 2011 | (Ojha et al., 2012) |
| Trivandrum (TRV) | Coastal, rural | 8.55 | 77.00 | 5 | 2007 – 2009 | (David et al., 2011) |
| Udaipur (UDP) | Urban | 24.58 | 73.68 | 598 | 2010 – 2011 | (Yadav et al., 2014) |

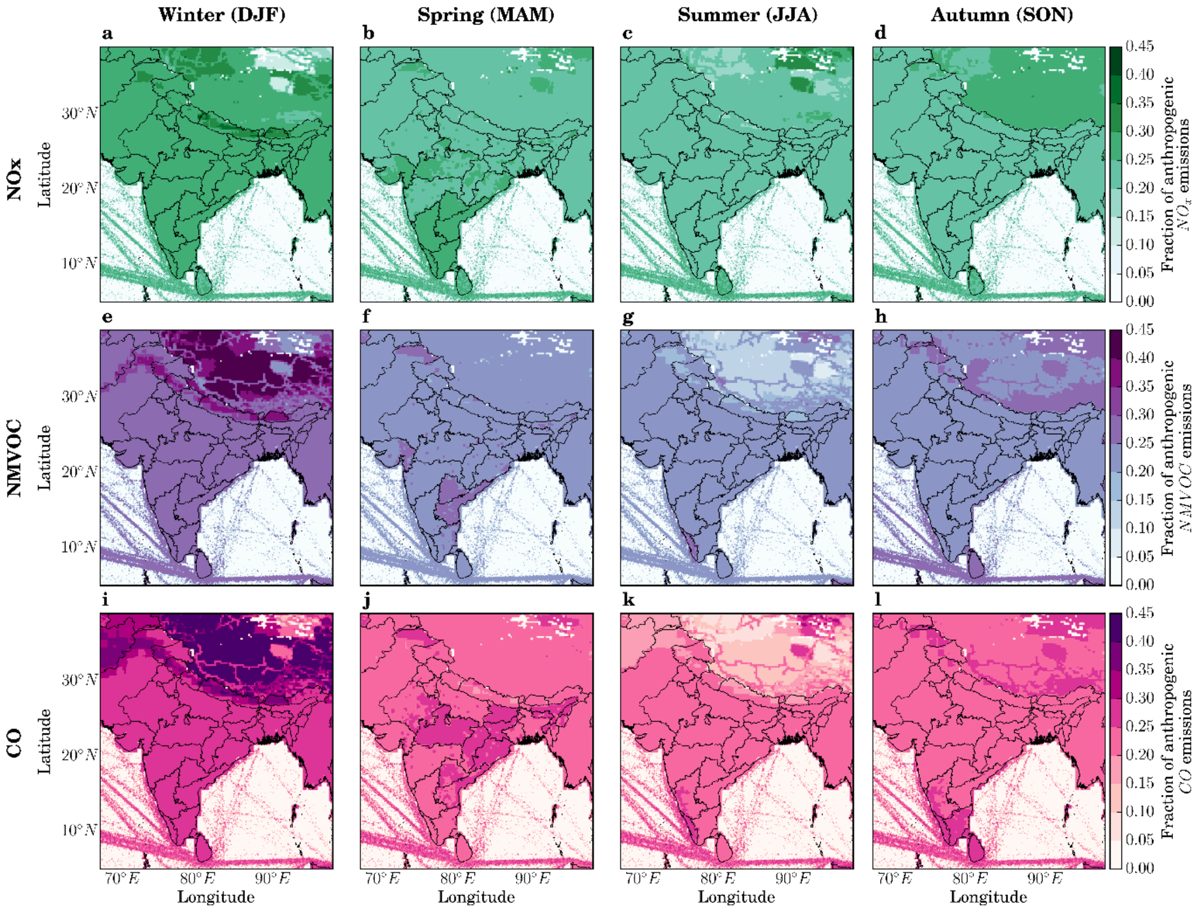


**Supplementary Figure 1:** Fractional contribution per season to total anthropogenic emissions. Fractional contribution to total anthropogenic emissions of NOx from winter (DJF), spring (MAM), summer (JJA), and autumn (SON) to anthropogenic emissions of (a – d) nitrogen oxides (NOx), (e – h) non-methane volatile organic compounds (NMVOC), and (i – l) carbon monoxide (CO).

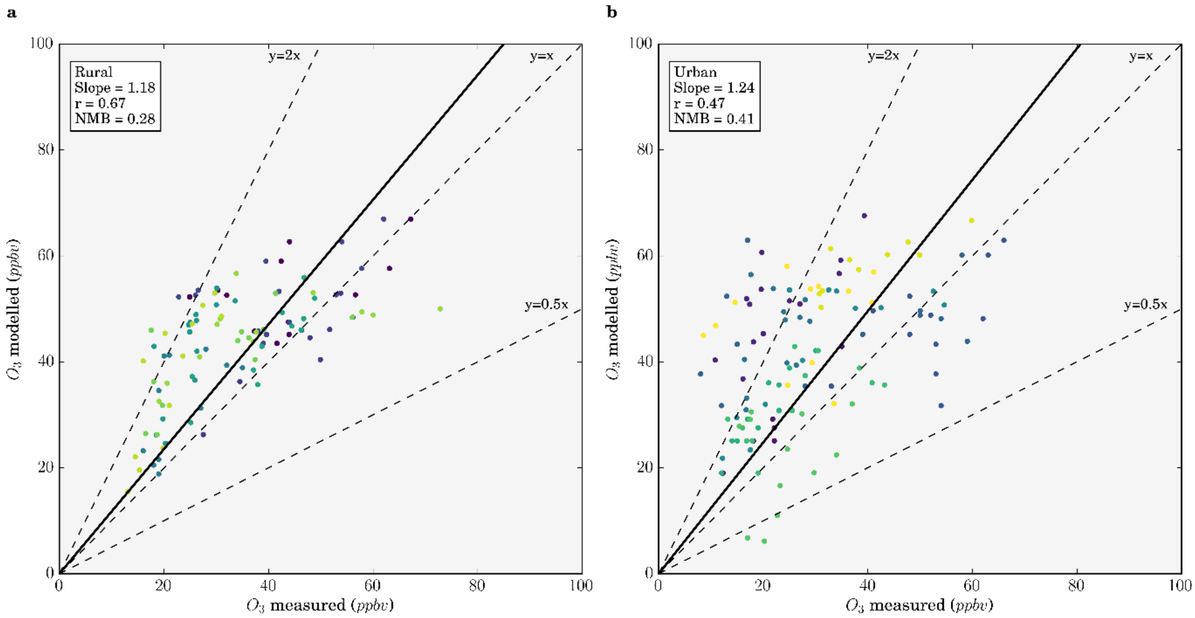


**Supplementary Figure 2:** Comparison of rural and urban observed and simulated $O_3$ concentrations. (a) Comparison of annual and monthly-mean ambient surface $O_3$ concentrations from rural observation sites. We show the rural site best fit line as solid, and the 1:1, 2:1, and 1:2 lines as dashed. Rural site normalised mean bias (NMB) = 0.28, the rural site best-fit line has slope = 1.18, and rural site Pearson's correlation coefficient (r) = 0.67. (b) Comparison of annual and monthly-mean ambient surface $O_3$ concentrations from urban observation sites. We show the urban site best fit line as solid, and the 1:1, 2:1, and 1:2 lines as dashed. Urban site NMB = 0.41; the urban site best-fit line has slope = 1.24, and urban site r = 0.47. Colours of filled circles grouped by site.

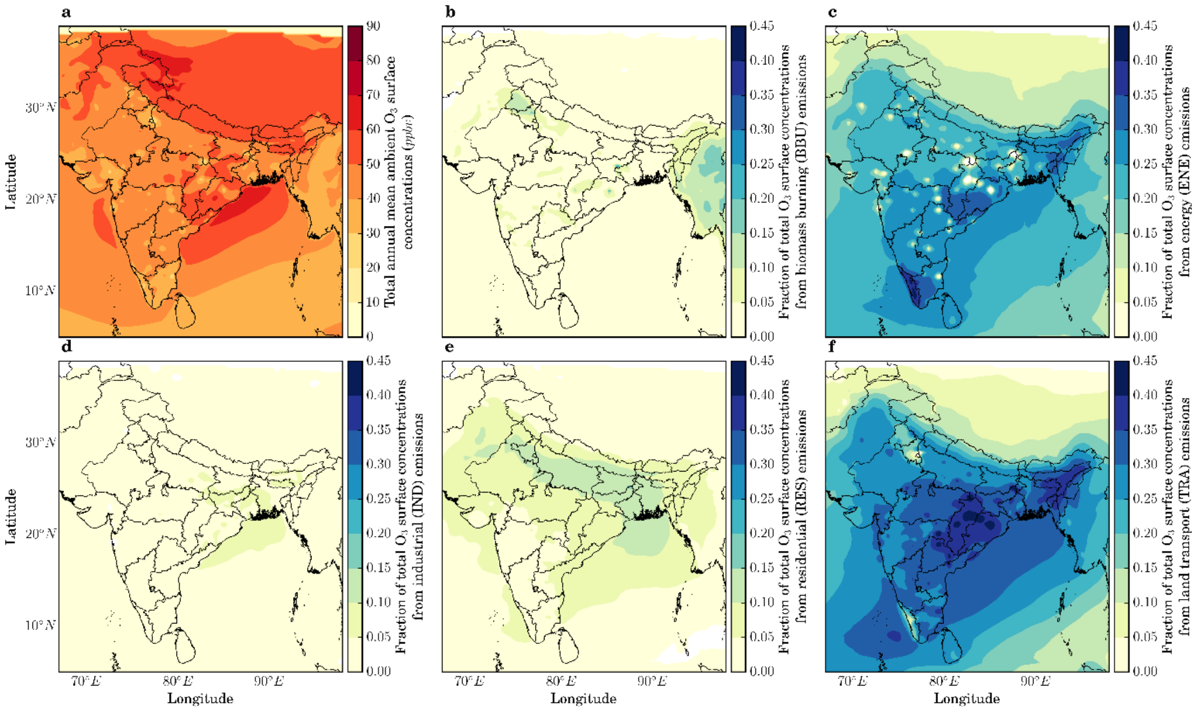


**Supplementary Figure 3:** Fractional contribution per source to total annual-mean ambient $O_3$ surface concentrations. (a) Total annual-mean ambient $O_3$ surface concentrations. (b – f) Fractional contribution from biomass burning (BBU), power generation (ENE), industrial non-power (IND), residential energy use (RES), and land transport (TRA).

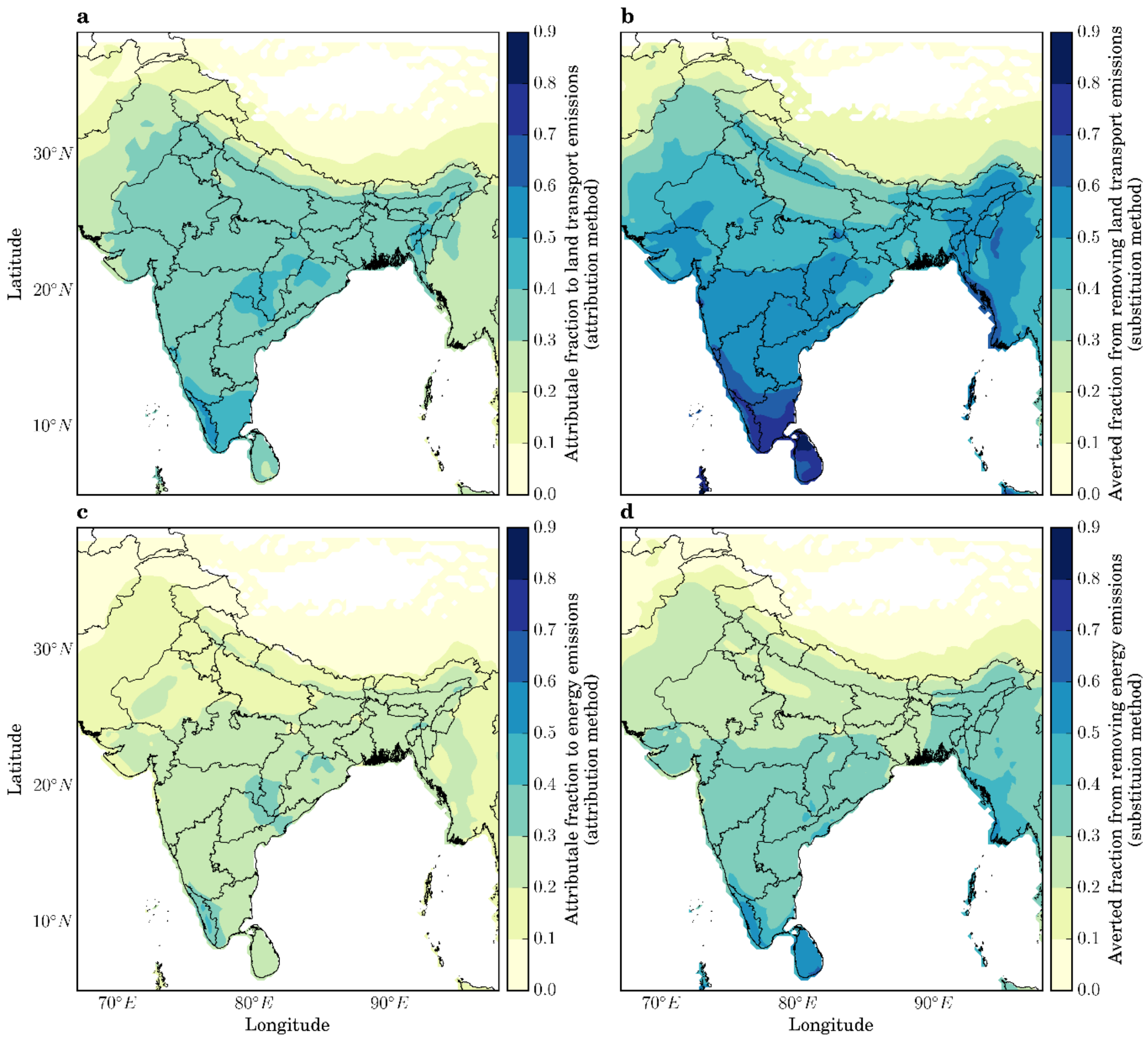


**Supplementary Figure 4:** Dominant source contributions to premature mortality burden due to $O_3$ exposure across India in 2015. (a) Attributable fraction of premature mortalities from land transport emissions (attribution method). (b) Averted fraction of premature mortalities from removing land transport emissions (subtraction method). (c) Attributable fraction of premature mortalities from energy emissions (attribution method). (d) Averted fraction of premature mortalities from removing energy emissions (subtraction method). All health impacts are calculated using Turner et al., (2016) RR and $LCC_{min}$.

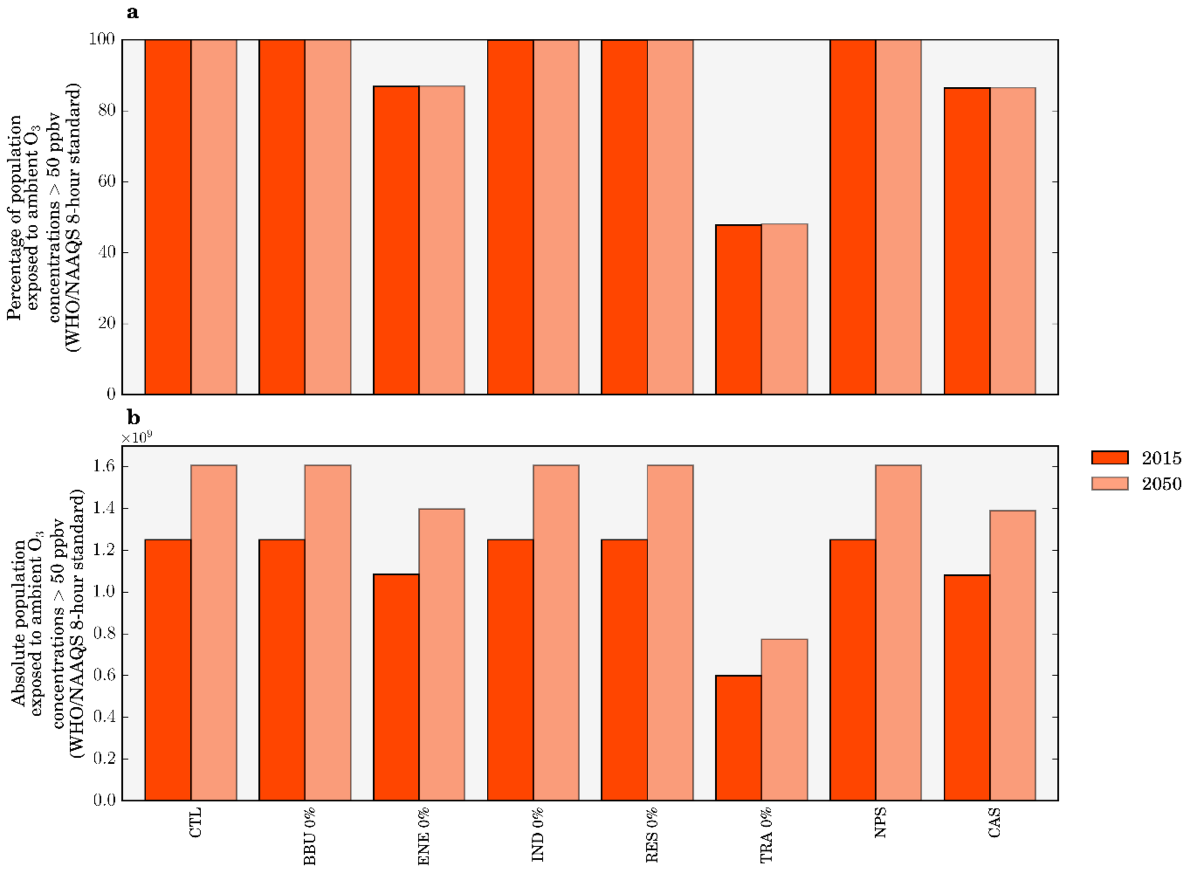


**Supplementary Figure 5:** The impact of scenarios on $O_3$ metrics. (a) Percentage of population in 2015 ($1^{st}$ bar) and 2050 ($2^{nd}$ bar) exposed to population-weighted ambient surface $O_3$ concentrations above 50 ppb (WHO AQG, Indian NAAQS) in each scenario. (b) Absolute population in 2015 ($1^{st}$ bar) and 2050 ($2^{nd}$ bar) exposed to population-weighted ambient surface $O_3$ concentrations above 50 ppb (WHO AQG, Indian NAAQS) in each scenario.

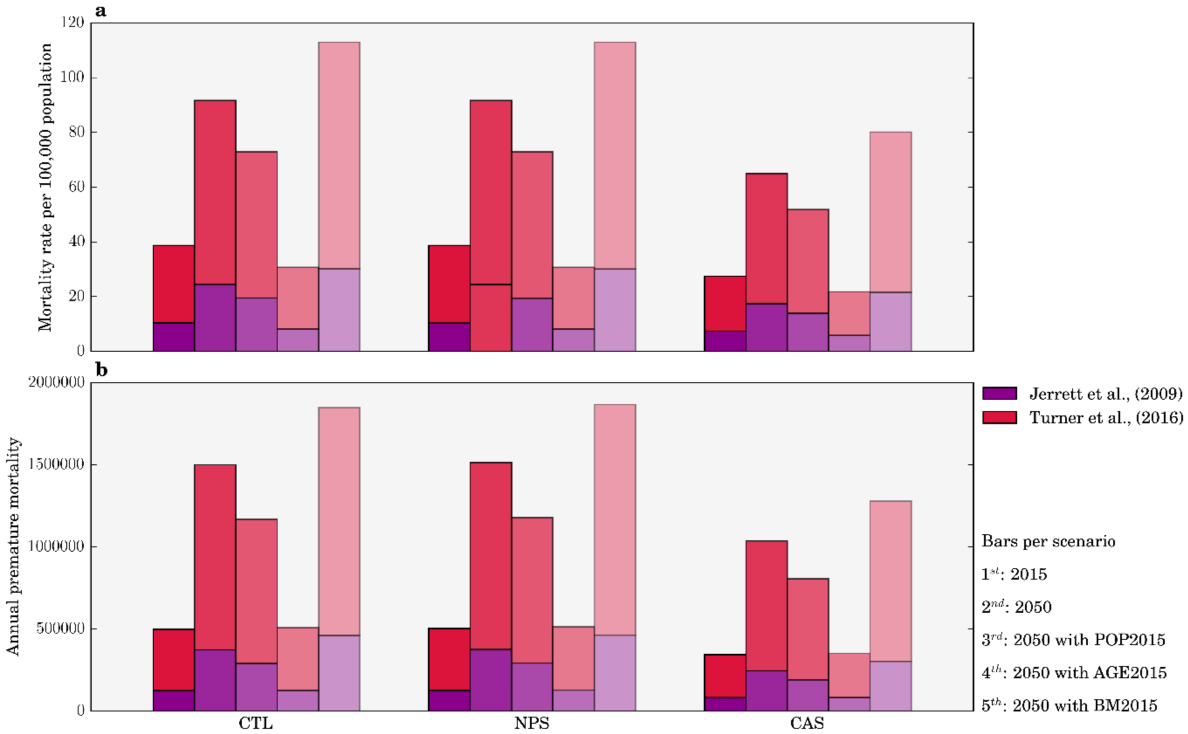


**Supplementary Figure 6:** Sensitivities of health impacts due to $O_3$ exposure in India to demography and baseline mortality rates. (a) Mortality rate per 100,000 population. (b) Total annual premature mortality. Impacts are estimated using either Jerrett et al., (2009) (red) and Turner et al., (2016) (purple) relative risks with $LCC_{min}$. For each panel, the control (CTL) scenario is compared against the NPS and CAS scenarios. For each panel, the five bars (left to right) show estimates for 2015 with 2015 population, age, and baseline mortality, 2050 with 2050 population, age, and baseline mortality, and 2050 with population from 2015 (POP2015), population age grouping from 2015 (AGE2015), and baseline mortality rates from 2015 (BM2015).

## References


Beig, G., Gunthe, S., & Jadhav, D. B. (2007). Simultaneous measurements of ozone and its precursors on a diurnal scale at a semi urban site in India. *Journal of Atmospheric Chemistry*, *57*(3), 239–253. https://doi.org/10.1007/s10874-007-9068-8

Chin, M., Ginoux, P., Kinne, S., Torres, O., Holben, B. N., Duncan, B. N., et al. (2002). Tropospheric Aerosol Optical Thickness from the GOCART Model and Comparisons with Satellite and Sun Photometer Measurements. *Journal of the Atmospheric Sciences*, *59*(3), 461–483. https://doi.org/10.1175/1520-0469(2002)059

Chin, M., Rood, R. B., Lin, S.-J., Müller, J.-F., & Thompson, A. M. (2000). Atmospheric sulfur cycle simulated in the global model GOCART: Model description and global properties. *Journal of Geophysical Research*, *105*(D20), 24671–24687. https://doi.org/10.1029/2000JD900384

David, L. M., & Nair, P. R. (2011). Diurnal and seasonal variability of surface ozone and NOx at a tropical coastal site: Association with mesoscale and synoptic meteorological conditions. *Journal of Geophysical Research*, *116,* D10303. https://doi.org/10.1029/2010JD015076

Ek, M. B., Mitchell, K. E., Lin, Y., Rogers, E., Grunmann, P., Koren, V., et al. (2003). Implementation of Noah land surface model advances in the National Centers for Environmental Prediction operational mesoscale Eta model. *Journal of Geophysical Research: Atmospheres*, *108*(D22), 8851–8867. https://doi.org/10.1029/2002JD003296

Emmons, L. K., Walters, S., Hess, P. G., Lamarque, J.-F., Pfister, G. G., Fillmore, D., et al. (2010). Description and evaluation of the Model for Ozone and Related chemical Tracers, version 4 (MOZART-4). *Geoscientific Model Development*, *3*, 43–67. https://doi.org//10.5194/gmd-3-43-2010

Gaur, A., Tripathi, S. N., Kanawade, V. P., Tare, V., & Shukla, S. P. (2014). Four-year measurements of trace gases (SO2, NOx, CO, and O3) at an urban location, Kanpur, in Northern India. *Journal of Atmospheric Chemistry*, *71*(4), 283–301. https://doi.org/10.1007/s10874-014-9295-8

Grell, G. A., & Devenyi, D. (2002). A generalized approach to parameterizing convection combining ensemble and data assimilation techniques. *Geophysical Research Letters*, *29*(14), 10–13. https://doi.org/10.1029/2002GL015311

Hodzic, A., & Knote, C. (2014). WRF-Chem 3.6.1: MOZART gas-phase chemistry with MOSAIC aerosols. *Atmospheric Chemistry Division (ACD), National Center for Atmospheric Research (NCAR),* 7.

Jain, S. L., Arya, B. C., Kumar, A., Ghude, S. D., & Kulkarni, P. S. (2005). Observational study of surface ozone at New Delhi, India. *International Journal of Remote Sensing*, *26*(16), 3515–3524. https://doi.org/10.1080/01431160500076616

Jerrett, M., Burnett, R. T., Pope, C. A., Ito, K., Thurston, G., Krewski, D., et al. (2009). Long-Term Ozone Exposure and Mortality. *New England Journal of Medicine, 360*(11), 1085–1095. https://doi.org/10.1056/NEJMoa0803894

Kumar, R., Naja, M., Venkataramani, S., & Wild, O. (2010). Variations in surface ozone at Nainital: A high-altitude site in the central Himalayas. *Journal of Geophysical Research: Atmospheres*, *115*(16), 1–12. https://doi.org/10.1029/2009JD013715

Lal, S., Naja, M., & Subbaraya, B. H. (2000). Seasonal variations in surface ozone and its

precursors over an urban site in India. *Atmospheric Environment*, *34*(17), 2713–2724. https://doi.org/10.1016/S1352-2310(99)00510-5

Mahapatra, P. S., Panda, S., Walvekar, P. P., Kumar, R., Das, T., & Gurjar, B. R. (2014). Seasonal trends, meteorological impacts, and associated health risks with atmospheric concentrations of gaseous pollutants at an Indian coastal city. *Environmental Science & Pollution Research*, *21*(19), 11418–11432. https://doi.org/10.1007/s11356-014-3078-2

Mallik, C., Lal, S., & Venkataramani, S. (2015). Trace gases at a semi-arid urban site in western India: Variability and inter-correlations. *Journal of Atmospheric Chemistry*, *72*(2), 143–164. https://doi.org/10.1007/s10874-015-9311-7

Mlawer, E. J., Taubman, S. J., Brown, P. D., Iacono, M. J., & Clough, S. A. (1997). Radiative transfer for inhomogeneous atmospheres: RRTM, a validated correlated-k model for the longwave. *Journal of Geophysical Research*, *102*(D14), 16663–16682. https://doi.org/10.1029/97JD00237

Naja, M., & Lal, S. (2002). Surface ozone and precursor gases at Gadanki (13.5°N, 79.2°E), a tropical rural site in India. *Journal of Geophysical Research: Atmospheres*, *107*(14). https://doi.org/10.1029/2001JD000357

Naja, M., Lal, S., & Chand, D. (2003). Diurnal and seasonal variabilities in surface ozone at a high altitude site Mt Abu (24.6°N, 72.7°E, 1680 m asl) in India. *Atmospheric Environment*, *37*(30), 4205–4215. https://doi.org/10.1016/S1352-2310(03)00565-X

Nakanishi, M., & Niino, H. (2006). An improved Mellor-Yamada Level-3 model: Its numerical stability and application to a regional prediction of advection fog. *Boundary-Layer Meteorology*, *119*(2), 397–407. https://doi.org/10.1007/s10546-005-9030-8

National Centers for Environmental Prediction, National Weather Service, National Oceanic and Atmospheric Administration, & U.S. Department of Commerce. (2000). NCEP Final (FNL) Operational Model Global Tropospheric Analyses, continuing from July 1999. Research Data Archive at the National Center for Atmospheric Research, Computational and Information Systems Laboratory. https://doi.org/http://dx.doi.org/10.5065/D6M043C6.

National Centers for Environmental Prediction, National Weather Service, National Oceanic and Atmospheric Administration, & U.S. Department of Commerce. (2007). NCEP Global Forecast System (GFS) Analyses and Forecasts. Research Data Archive at the National Center for Atmospheric Research, Computational and Information Systems Laboratory. https://doi.org/http://rda.ucar.edu/datasets/ds084.6/

Nishanth, T., Satheesh Kumar, M. K., & Valsaraj, K. T. (2012). Variations in surface ozone and NOx at Kannur: a tropical, coastal site in India. *Journal of Atmospheric Chemistry*, *69*(2), 101–126. https://doi.org/10.1007/s10874-012-9234-5

Ojha, N., Naja, M., Singh, K. P., Sarangi, T., Kumar, R., Lal, S., et al. (2012). Variabilities in ozone at a semi-urban site in the Indo-Gangetic Plain region: Association with the meteorology and regional processes. *Journal of Geophysical Research: Atmospheres*, *117*(20), 1–19. https://doi.org/10.1029/2012JD017716

Pincus, R., Barker, H. W., & Morcrette, J.-J. (2003). A fast, flexible, approximate technique for computing radiative transfer in inhomogeneous cloud fields. *Journal of Geophysical Research*, *108*(D13), 1–5. https://doi.org/10.1029/2002JD003322

Reddy, B. S. K., Kumar, K. R., Balakrishnaiah, G., Gopal, K. R., Reddy, R. R., Ahammed, Y. N., et al. (2010). Observational studies on the variations in surface ozone concentration at

Anantapur in southern India. *Atmospheric Research, 98*(1), 125–139. https://doi.org/10.1016/j.atmosres.2010.06.008

Reddy, R. R., Gopal, K. R., Reddy, L. S. S., Narasimhulu, K., Kumar, K. R., Ahammed, Y. N., et al. (2008). Measurements of surface ozone at semi-arid site Anantapur (14.62 degrees N, 77.65 degrees E, 331 m asl) in India. *Journal of Atmospheric Chemistry, 59*(1), 47–59. https://doi.org/10.1007/s10874-008-9094-1

Sahu, L. K., & Lal, S. (2006). Distributions of C2-C5 NMHCs and related trace gases at a tropical urban site in India. *Atmospheric Environment*, *40*(5), 880–891. https://doi.org/10.1016/j.atmosenv.2005.10.021

Sarangi, T., Naja, M., Ojha, N., Kumar, R., Lal, S., Venkataramani, S., et al. (2014). First simultaneous measurements of ozone, CO, and NOy at a high-altitude regional representative site in the central Himalayas. *Journal of Geophysical Research: Atmospheres*, *19*, 1592–1611. https://doi.org/10.1002/2013JD020631

Sarkar, S., Srivastava, R. K., & Sagar, K. (2015). Diurnal Monitoring Of Surface Ozone And PM2.5 Concentration And Its Correlation With Temperature. *INTERNATIONAL JOURNAL OF TECHNOLOGY ENHANCEMENTS AND EMERGING ENGINEERING RESEARCH*, *3*(09), 121–129.

Sharma, P., Kuniyal, J. C., Chand, K., Guleria, R. P., Dhyani, P. P., & Chauhan, C. (2013). Surface ozone concentration and its behaviour with aerosols in the northwestern Himalaya, India. *Atmospheric Environment*, *71*, 44–53. https://doi.org/10.1016/j.atmosenv.2012.12.042

The National Center for Atmospheric Research. (2016). ACOM MOZART-4/GEOS-5 global model output. *UCAR*.

Thompson, G., Rasmussen, R. M., & Manning, K. (2008). Explicit Forecasts of Winter Precipitation Using an Improved Bulk Microphysics Scheme. Part II: Implementation of a New Snow Parameterization. *American Meteorological Society*, *136*(2), 5095–5115. https://doi.org/10.1175/2008MWR2387.1

Tie, X., Madronich, S., Walters, S., Zhang, R., Rasch, P., & Collins, W. (2003). Effect of clouds on photolysis and oxidants in the troposphere. *Journal of Geophysical Research*, *108*(D20), 4642, 1–11. https://doi.org/10.1029/2003JD003659

Turner, M. C., Jerrett, M., Pope III, C. A., Krewski, D., Gapstur, S. M., Diver, R. W., et al. (2016). Long-Term Ozone Exposure and Mortality in a Large Prospective Study. *American Journal of Respiratory and Critical Care Medicine*, *193*(10), 1134–1142. https://doi.org/10.1164/rccm.201508-1633OC

Yadav, R., Sahu, L. K., Jaaffrey, S. N. A., & Beig, G. (2014). Distributions of ozone and related trace gases at an urban site in western India. *Journal of Atmospheric Chemistry*, *71*(2), 125–144. https://doi.org/10.1007/s10874-014-9286-9

Zaveri, R. A., Easter, R. C., Fast, J. D., & Peters, L. K. (2008). Model for Simulating Aerosol Interactions and Chemistry (MOSAIC). *Journal of Geophysical Research*, *113*(D13204), 1–29. https://doi.org/10.1029/2007JD008782
